# Long-distance, synchronized and directional fall movements suggest migration in Arctic hares on Ellesmere Island (Canada)

**DOI:** 10.1038/s41598-022-08347-1

**Published:** 2022-03-23

**Authors:** Jacob Caron-Carrier, Sandra Lai, François Vézina, Andrew Tam, Dominique Berteaux

**Affiliations:** 1 Département de biologie, chimie et géographie, Université du Québec à Rimouski, 300 Allée des Ursulines, Rimouski, QC G5L 3A1 Canada; 2 Canada Research Chair on Northern Biodiversity, Université du Québec à Rimouski, 300 Allée des Ursulines, Rimouski, QC G5L 3A1 Canada; 3 Centre for Northern Studies, Université du Québec à Rimouski, 300 Allée des Ursulines, Rimouski, QC G5L 3A1 Canada; 4 Quebec Centre for Biodiversity Science, Université du Québec à Rimouski, 300 Allée des Ursulines, Rimouski, QC G5L 3A1 Canada; 5 Department of National Defence, 8 Wing Canadian Forces Base Trenton, P.O. Box 1000, Station Forces, Astra, ON K0K 3W0 Canada

**Keywords:** Behavioural ecology, Animal migration, Ecology, Animal behaviour

## Abstract

Animal migration contributes largely to the seasonal dynamics of High Arctic ecosystems, linking distant habitats and impacting ecosystem structure and function. In polar deserts, Arctic hares are abundant herbivores and important components of food webs. Their annual migrations have long been suspected, but never confirmed. We tracked 25 individuals with Argos satellite telemetry to investigate the existence of migration in a population living at Alert (Ellesmere Island, Nunavut, Canada). During fall, 21 hares undertook directional, long-distance movements in a southwestern direction towards Lake Hazen. Daily movement rates averaged 1.3 ± 0.5 km, 4.3 ± 1.6 km, and 1.7 ± 0.9 km before, during, and after relocation, respectively. Straight-line and minimum cumulative distances traveled averaged 98 ± 18 km (range: 72–148 km) and 198 ± 62 km (range: 113–388 km), respectively. This is the first report of large-scale seasonal movements in Arctic hares and, surprisingly, in any lagomorph species. These movements may be part of an annual migratory pattern. Our results redefine our understanding of the spatial ecology of Arctic hares, demonstrate unsuspected mobility capacities in lagomorphs, and open new perspectives regarding the ecological dynamics of the northern polar deserts.

## Introduction

Long-distance movements of animals are widespread and have far-reaching implications for the dynamics of ecosystems^1–3^. For example, individuals moving across ecosystem boundaries may transport nutrients, parasites, and pathogens^1,4,5^. The mass arrival of new animals in a system may also alter local food webs by introducing new predators^6^ or prey^7^. Identifying the extent of movements exhibited by species or populations can therefore provide critical ecological insight and yield new knowledge pertinent to management or conservation^8^.

Three broad categories of long-distance movements are generally recognized^9^. Dispersal occurs when an animal leaves a previously used area to move to another area that will be used for breeding^10^. Migration is a periodic movement between two distinct areas, such as a summer and a winter range^10,11^. Finally, nomadism involves movements that may be highly variable in their timing and direction within and between years^10,11^.

Migration and nomadism are common in strongly seasonal environments, since moving animals can benefit from tracking the fluctuations of resources and weather^10,12^. Northern polar deserts represent extreme environments defined by low temperatures and precipitation^13,14^ which, combined with nutrient-poor soils, result in low plant productivity^15,16^. Winters are extremely cold (temperatures routinely reach − 40 °C), and access to vegetation may be reduced by hard layers of snow such as hard wind slabs and melt-freeze crusts^17^. To cope with the high seasonality of this environment, Arctic species use multiple movement strategies, ranging from range residency^18^ to migration^19^ and nomadism^20^. While the long migrations of Arctic birds are well known, at least 18 of the 70 species of Arctic terrestrial mammals also have migratory individuals^21^. More generally, migration is diffusely spread throughout the Mammal class (found in 12 of 27 orders)^22^, with concentrations of migratory species within Cetacea, Artiodactyla, and Chiroptera^12,22^, and suggested benefits belonging to four categories: increased energy intake, decreased energy expenditure, decreased predation, and increased mating opportunities^12^.

The Arctic hare (*Lepus arcticus*) is one of the largest lagomorphs and an important component of the polar desert food web^23^. It is omnipresent in the diet of multiple predators, such as Arctic wolves (*Canis lupus arctos*)^24,25^ and Arctic foxes (*Vulpes lagopus*)^23^. Arctic hares are also generalist herbivores that are active year-round and can reach high densities^23^. Their movements may therefore influence both upper (predators) and lower (plants) levels of the High Arctic food web. While the species range encompasses the whole Canadian Arctic and a portion of coastal Greenland, their spatial ecology has only been investigated in Newfoundland, Canada^26–28^. A recurrent debate about Arctic hare ecology at the highest latitudes is whether they migrate seasonally. Harper^29^ observed in southwestern Keewatin (Nunavut), that hares disappeared during the summer and returned in November. While Harper^29^ believed that Arctic hares migrated, no empirical evidence supports this claim and Dalerum et al.^30^ recently questioned whether lagomorphs possess the locomotion abilities required to accomplish large-scale movements. Current literature suggests that Arctic hares are a sedentary species with little dispersal capacity^28^.

Despite the extraordinary techniques now available to track wildlife, Arctic hare movements have never been studied in the High Arctic. Very little is therefore known on this topic, mostly due to the logistic constraints associated with capturing many individuals in difficult-to-reach locations. In addition to increasing our understanding of navigation and locomotion in animals, our improved ability to track individuals precisely and over long periods has facilitated the integration of movement ecology with wildlife management and conservation^31^. While rapid global changes significantly alter landscapes at large scales, species status assessments increasingly use data on species mobility to evaluate conservation threats^31^. Currently, Arctic hares have no legal conservation status in Canada due to lack of data, but their range is restricted to the Arctic, a region strongly exposed to climate change and increasing human activities^32^. Better knowledge of Arctic hare movements is therefore necessary to close knowledge gaps regarding both their movement ecology and their conservation.

Here, we used satellite tracking to investigate seasonal movements of Arctic hares on Ellesmere Island, Nunavut, in the polar desert of the Canadian High Arctic. Our objectives were to (i) test the hypothesis that migration occurs in an Arctic hare population at the species northern distribution limit, (ii) characterize seasonal movement metrics, including timing of movements, and (iii) identify areas of seasonal residency. We report relatively synchronized and directional long-distance movements of Arctic hares during the fall. The scale of these movements, reaching several hundred kilometers, has never been observed in any lagomorph species.

## Methods

### Study area

**Figure 1:**
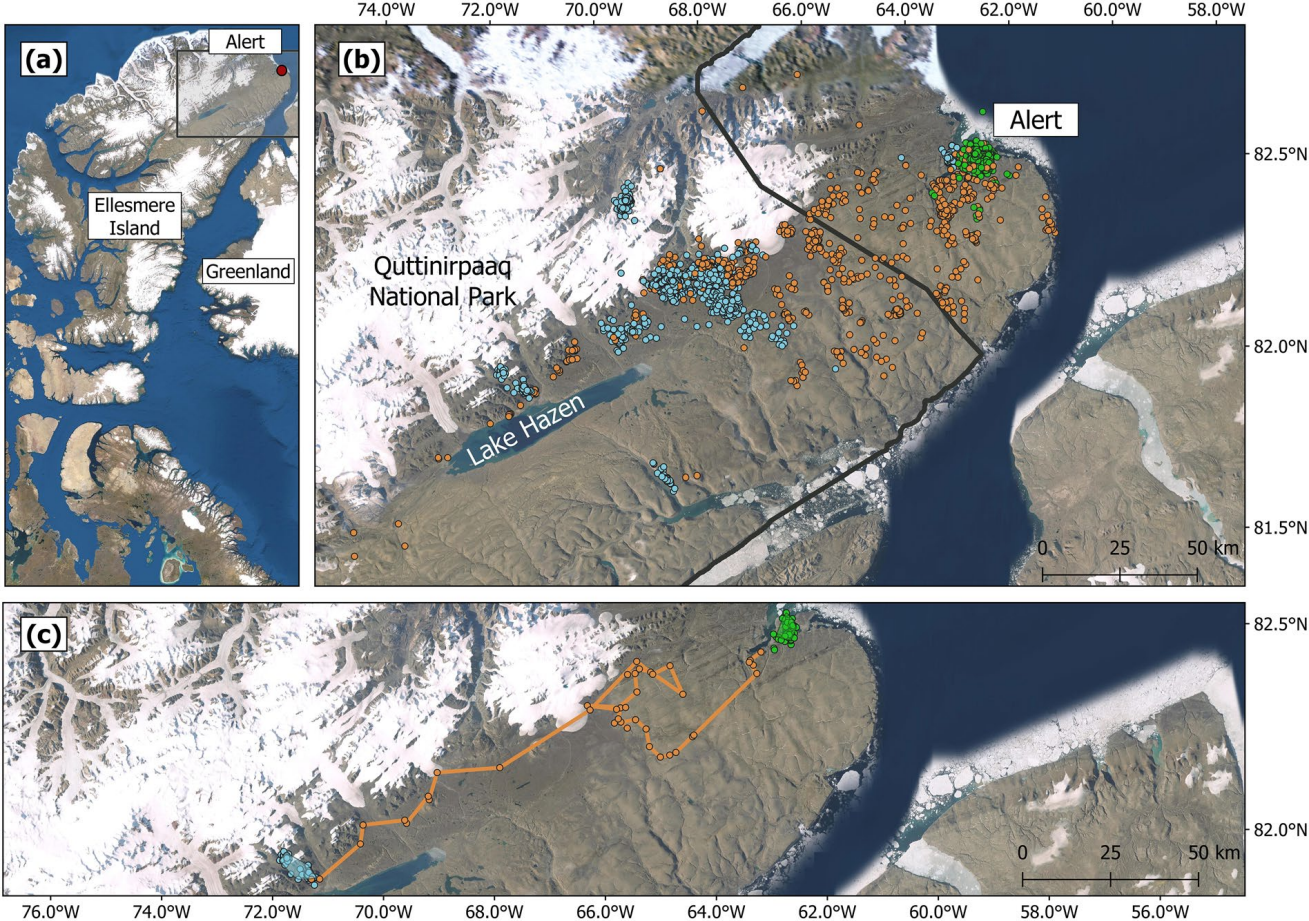
Location of study area and spatial extent of Arctic hare movements at the northeastern tip of Ellesmere Island. (**a**) Location of Alert (red dot) on Ellesmere Island, west of Greenland. The grey rectangle is enlarged in (**b**). (**b**) Locations of 25 hares, obtained by Argos telemetry between 15 June 2019–31 May 2020, are represented by green circles (summer locations), orange circles (fall relocation), and blue circles (winter locations). (**c**) Summer range (green), relocation path (orange dots and line) and winter range (blue) of an Arctic hare collared at Alert and wintering in the Lake Hazen area. The boundaries of the Quttinirpaaq National Park of Canada are represented by a black line. This map was created using QGIS 3.8.3^40^. Satellite imagery was obtained from: Esri, USGS | Esri, HERE, Garmin, FAO, NOAA, USGS, NRCan, Parks Canada | Earthstar Geographics.

We worked in a 170-km^2^ study area surrounding Canadian Forces Station Alert, Ellesmere Island, Nunavut (82°30′N, 62°20′W; Fig. 1a). The landscape encompasses multiple hills and creeks, four lakes, and several ponds^33^. With only 156 mm of precipitation per year on average, and temperatures reaching − 40 °C in winter, the environment is a polar desert^33^. Due to the short growing season (60–70 days) and dry soils poor in nutrients, only 77 species of vascular plants grow at Alert^33^. The most common species are saxifrages (*Saxifraga *sp.), Arctic poppies (*Papaver *sp*.*), Arctic willows (*Salix arctica*), and several species of grasses^33^. The main herbivores at Alert are the Arctic hare, the collared lemming (*Dicrostonyx groenlandicus*), the muskox (*Ovibos moschatus*), the Peary caribou (*Rangifer tarandus pearyi*), and the rock ptarmigan (*Lagopus muta*). At Alert, predators of young and adult hares include the Arctic wolf, the Arctic fox, and the snowy owl (*Bubo scandiacus*). Ermines (*Mustela erminea*) and glaucous gulls (*Larus hyperboreus*) may also predate leverets at Alert.

### Captures and satellite tracking

**Figure 2:**
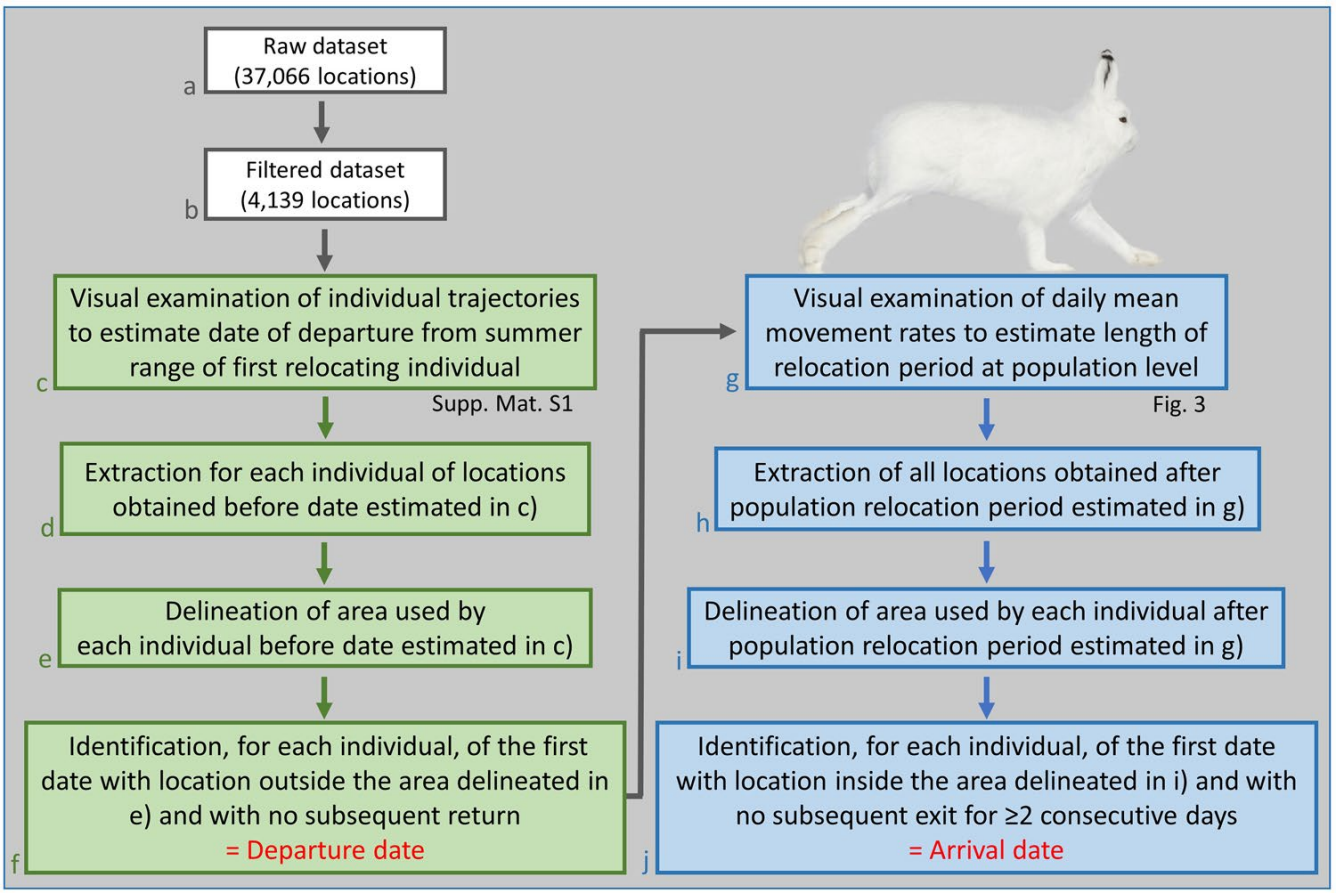
Methodological workflow for the identification of fall departure and arrival dates of Arctic hares tracked at Alert (Nunavut, Canada). After acquisition and filtering of location data (steps a–b, white boxes), departure dates (steps c–f, green boxes) and arrival dates (steps g–j, blue boxes) were obtained through a multi-step process involving visualization of individual trajectories and delineation of individual summer and winter grounds.

Arctic hares were captured using Tomahawk cage traps (102 cm × 38 cm × 38 cm, model 208, Tomahawk Live Trap Co, Tomahawk, WI, USA) and custom-made drop cages (95 cm × 95 cm × 45 cm). Cages were baited with peanuts and commercial bird seeds and checked every 2–4 h. Capture effort occurred from 15 May to 26 July 2019 and was irregular through space and time, depending on weather conditions, opportunistic hare observations, and time constraints. Upon capture, we determined sex, mass (nearest 50 g, Pesola spring scale 10 kg), age class based on body size (juvenile or ≥ 1 year old, the latter being referred to as “adult”) and, for females, reproductive status (pregnant, lactating, or undetermined). Pregnancy was assessed by examination and palpation of the abdomen, and lactation by squeezing milk from the teats^34^. Adults were ear-tagged using four numbered metal bands (Jiffy Wing Bands—Style 893, National Band & Tag Company) to which were attached custom-made plastic color tags (1.5 cm × 2.5 cm) providing unique color codes allowing individual identification at a distance or during recaptures. From 14 June to 26 July 2019, 25 hares were fitted with an Argos Platform Terminal Transmitter (PTT, model KiwiSat 303, Lotek, Newmarket, Ontario, Canada; 115 g; 2–3.1% of body mass) with a temperature sensor. Capture and handling techniques were approved by the Animal Care Committee of Université du Québec à Rimouski (CAC-68-17-184) and the Government of Nunavut (Permit number WL 2018-020). The dataset generated and analyzed for this study (Fig. 2a) is part of the Arctic Animal Movement Archive^35^, is freely available in MoveBank^36^, and is stored in the MoveBank Data Repository^37^.

### Argos location filter

PTT collars were programmed to transmit daily between 10:00–13:00 (local time) with a repetition rate of 60 s. To maximize both accuracy and number of days with locations for each hare, we filtered positions (Fig. 2b) with a Location Class 3, 2, 1, and A, using a speed filter implemented in R 3.6.2^38^ (see S1 in Christin et al.^39^). The speed filter removed locations that were likely erroneous based on a > 5 km/h cruising speed, with possible acceleration bouts of 10 km/h for locations less than 10 min apart. We set these values based on preliminary data analysis. We then kept for further analyzes one location per day per hare, based on the smallest location error. A visual check of the data confirmed that no outlier locations remained.

### Determination of departure date from summer ground

Our visual inspection of individual trajectories (QGIS 3.8.3^40^, Fig. 2c) showed that no hare left its summer grounds before 11 August. We subsequently determined departure dates of individuals in two steps. First, we delineated for each hare the 95% minimum convex polygon (MCP) of the area used after collaring but before 11 August (Fig. 2d,e). For most individuals, locations were too few (median: 32, range: 12–54) for this area to reach an asymptotic size^41^ and be considered a home range. Second, we identified the first day with a location outside of, and with no subsequent return to this area. We identified this day as the departure date from summer grounds (Fig. 2f). All hares departing then traveled > 80 km, which prevented any ambiguity regarding departure date. Hares that did not leave their summer grounds were classified as residents.

### Determination of arrival date on winter ground

**Figure 3:**
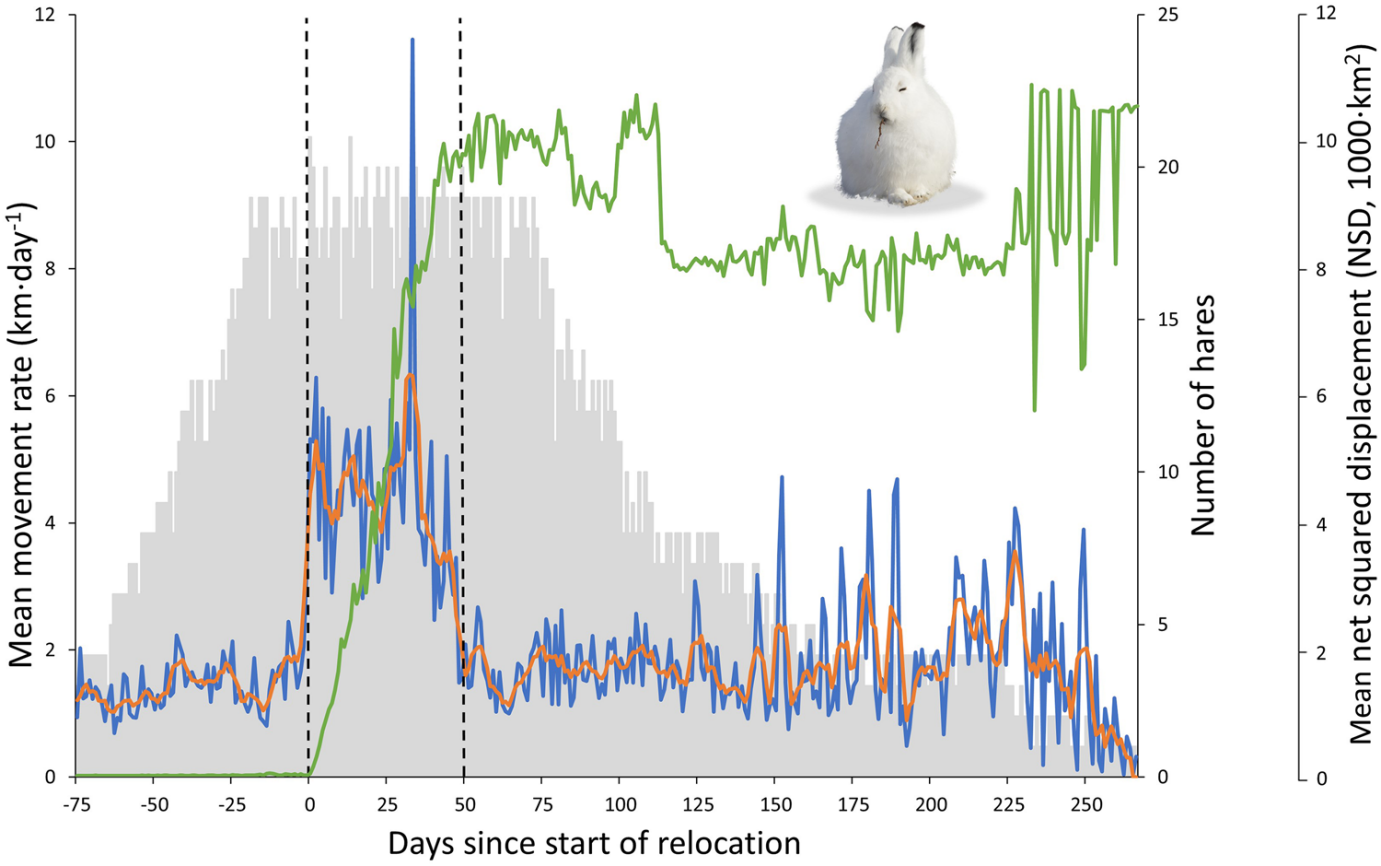
Variation through time of the mean movement rate and mean net squared displacement (NSD) of Arctic hares equipped with Argos satellite collars at Alert (Nunavut, Canada). Departure dates of individuals from their summer grounds ranged from 11 August 2019 to 17 September 2019 (on average 26 August 2019) and were considered as day 0 before plotting. Mean daily movement rates (blue line) and 5-day moving average of daily movement rates (orange line) are shown from day − 75 (on average 27 June 2019) to day 266 (on average 30 May 2020). Mean net squared displacement (NSD, green line) is shown for the same period. The number of hares contributing data to a given day is shown as a gray bar. Vertical dashed lines indicate the average start (day 0) and end (day 50, on average 15 October 2019) of relocation of tracked hares. Note the sharp increase of movement rates and NSD when relocation begins, but the more gradual change when relocation ends, due to individual differences in length of the relocation period.

Terrestrial migratory herbivores usually show much higher movement rates during migration than before or after migration^42–46^. We therefore expected that hares leaving their summer grounds would show high movement rates during relocation. We define here relocation as the action of moving to a new area, happening between departure from summer grounds and arrival to winter grounds. To confirm high movement rates during relocation, we calculated daily individual movement rates for the entire (filtered) dataset generated for each hare. Daily movement rates are defined as the straight-line distance between daily locations or, in rare cases when some daily locations were missing, the straight-line distance between locations divided by the number of days elapsed between locations. Accordingly, average daily movement rate of individuals dramatically increased after they departed from their summer grounds, remained high during ca. 50 days (the relocation period of the population), then returned to values observed during summer when most individuals had reached their winter grounds (see “Results”, Fig. 3). Using our estimate of the average length of the relocation period (Fig. 2g), we determined arrival dates of individuals on winter grounds in two steps. First, using locations collected > 50 days after their departure (Fig. 2h), we delineated for each relocated hare the 95% MCP used after the relocation period of the population (Fig. 2i). Second, we identified the arrival date of each hare on its winter grounds as the day it entered the above area and remained in it for ≥ 2 consecutive days (Fig. 2j).

### Determination and statistical testing of movement metrics describing hare residency and relocation

We used 10 variables to describe residency and relocation of Arctic hares (Table 1). Of those, three describe the timing of relocation: departure date from summer grounds, arrival date to winter grounds, duration of relocation (time elapsed between start and end of relocation). Four variables describe the traveling behaviour of hares: minimum cumulative traveled distance from start to end of tracking (sum of the distances between consecutive daily locations of a hare path), minimum cumulative relocation distance (same as above but from start to end of relocation), straight line distance between summer and winter grounds (minimum distance between centroids of summer and winter MCPs calculated below), orientation of relocation travel (bearing of the line joining the centroids of summer and winter MCPs calculated below). Finally, three surface variables describe areas used by tracked hares: size of summer and winter grounds for relocating hares (summer 95% MCP calculated from locations collected between collaring date and departure date, and winter 95% MCP calculated from locations collected between arrival date and end of tracking), and size of annual grounds for resident hares (95% MCP calculated over entire tracking period). Note that summer and winter MCPs were calculated for each hare using individual departure and arrival dates, and can thus slightly differ from MCPs calculated at steps e and i of Fig. 2. For each individual, we displayed the movement path, along with the net squared displacement (NSD) curve starting from the capture location (Supplementary material S1).

**Table 1:** Metrics describing individual Arctic hares and their movement ecology in a population studied at Alert (Nunavut, Canada). Timing of relocation and metrics describing travels and used areas are indicated for 25 hares tracked from 15 June 2019 to 31 May 2020. ID combines the sex of the individual (F, female; M, male), followed by a unique number. Ear-tag colors of individuals are also given in parentheses. ^1^Applies only to relocated individuals. ^2^Values in square brackets give sample size (number of locations).

Individuals				Timing of relocation			Travel metrics^2^						Used area metrics^2^					
ID (color code)	Reproductive status at capture	Mass at capture (kg)	Length of monitoring period (days)	Start	End	Duration (days)	Minimum total cumulative distance (km)		Minimum relocation cumulative distance (km)		Straight-line relocation distance (km)	Orientation of relocation (°)	Size of summer grounds (km2)		Size of winter grounds (km2)		Size of annual grounds (km^2^)	
F1 (GBVV)	Lactating	5.00	127	11 Aug	25 Sep	45	319 [117]		164 [40]		74	− 114.2	2 [24]		68 [53]		–	
F2 (YYYV)	Lactating	4.90	108	11 Aug	30 Sep	50	275 [103]		168 [46]		97	− 109.7	2 [26]		37 [31]		–	
F3 (BYRV)	Lactating	4.45	150	13 Aug	27 Sep	45	449 [132]		230 [38]		85	− 111.2	2 [12]		66 [82]		–	
F4 (VBRV)	Lactating	4.70	166	13 Aug	30 Sep	48	399 [149]		191 [44]		97	− 111.1	3 [17]		280 [88]		–	
F5 (YYRR)	Lactating	5.05	125	13 Aug	1 Oct	49	301 [125]		218 [49]		97	− 109.6	4 [27]		5 [49]		–	
F6 (BGGR)	Lactating	5.00	127	15 Aug	22 Sep	38	278 [106]		180 [28]		132	− 131.0	5 [35]		13 [43]		–	
F7 (BGVY)	Lactating	4.90	250	15 Aug	9 Oct	55	608 [218]		197 [52]		84	− 115.9	3 [16]		132 [150]		–	
F8 (BYYG)	Lactating	4.25	48	17 Aug	10 Sep	24	136 [32]		113 [17]		72	− 144.7	4 [15]		–		–	
F9 (RBBB)	Unknown	4.85	146	18 Aug	12 Oct	55	417 [130]		217 [46]		8	− 92.4	13 [42]		11 [42]		–	
F10 (RRYV)	Lactating	4.25	143	25 Aug	28 Sep	34	346 [113]		186 [29]		87	− 110.1	21 [21]		22 [63]		–	
F11 (GVRG)	Lactating	4.80	116	30 Aug	16 Oct	47	264 [115]		187 [47]		97	− 109.6	3 [42]		4 [26]		–	
F12 (YVVY)	Lactating	4.90	324	1 Sep	11 Oct	40	664 [282]		178 [35]		88	− 113.3	5 [56]		376 [191]		–	
M1 (YGRG)	–	3.70	143	1 Sep	12 Oct	41	341 [133]		171 [39]		95	− 109.7	9 [49]		18 [45]		–	
F13 (GBYR)	Lactating	4.30	192	2 Sep	1 Oct	29	364 [175]		161 [27]		93	− 107.9	7 [36]		36 [112]		–	
F14 (BVVY)	Lactating	4.50	221	2 Sep	11 Oct	39	593 [198]		188 [34]		100	− 110.5	61 [42]		358 [122]		–	
F15 (VVBB)	Unknown	4.10	301	3 Sep	11 Oct	38	560 [300]		172 [38]		85	− 113.5	5 [77]		165 [185]		–	
F16 (VRRB)	Unknown	4.60	177	4 Sep	18 Oct	44	472 [167]		266 [41]		148	− 111.4	27 [59]		38 [67]		–	
F17 (GGYY)	Unknown	4.60	341	7 Sep	22 Sep	15	523 [304]		140 [14]		103	− 92.9	12 [73]		17 [217]		–	
F18 (RGGG)	Lactating	4.40	149	12 Sep	1 Nov	50	415 [141]		310 [48]		112	− 111.3	5 [76]		3 [17]		–	
M2 (RGRG)	–	3.90	135	13 Sep	20 Oct	37	285 [127]		132 [35]		95	− 108.4	12 [52]		13 [40]		–	
F19 (BBYY)	Pregnant	5.80	165	17 Sep	5 Nov	49	557 [151]		388 [46]		119	− 112.4	13 [80]		9 [25]		–	
F20 (BBGG)	Pregnant	4.60	346	–	–	–	410 [332]		–		–	–	–		–		13 [332]	
F21 (RVBV)	Lactating	4.60	202	–	–	–	213 [202]		–		–	–	–		–		13 [202]	
M3 (BYBY)	–	3.90	225	–	–	–	310 [191]		–		–	–	–		–		20 [191]	
M4 (RYRY)	–	4.10	80	–	–	–	111 [71]		–		–	–	–		–		11 [71]	
Mean	–	4.57	180	26 Aug^1^	6 Oct^1^	42^1^	385 [4114]		198 [793]		94	− 111.9	10 [877]		83 [1648]		14 [796]	
SD	–	0.46	79	12	13	10	144		62		26	0.2	13		118		4	

We used a linear mixed-effect model in the lme4 package (version 1.1-27.1)^47^ in R to verify that movement rates differed significantly across the three movement phases (summer residency, fall relocation, and winter residency). We also included in the model the synchronised time as fixed effect and individual ID as random effect, to account for the unequal number of observations between individuals. We log-transformed (log10) movement rates to respect the assumptions of variance homogeneity and normality of residuals. Given that movement phase had a significant effect, we conducted a pairwise comparison between phases using Tukey’s method.

### Mapping of summer and winter grounds

We identified the summer and winter grounds at population level by creating kernel density surfaces using fixed kernel density estimation (KDE) with plug-in bandwidth selection (h_plug-in_) implemented in the ks package (version 1.13.0)^48^ in R (data projection: arctic polar stereographic; grid size: 500 × 500 m)^49,50^. To account for the varying number of locations per animal, we delineated summer grounds using the last 45 consecutive locations (corresponding to approximately 1.5 month of data) obtained before the start of autumn relocation for each hare. For resident hares, we included the last 45 consecutive locations collected before 11 August. Since 16 hares were captured less than 1.5 month before their departure date (or 11 August for residents), the mean number of locations used to delineate summer grounds was 35 ± 11 (median = 42, range: 12–45, n = 25). Similarly, we delineated winter grounds using the first 45 consecutive locations obtained after the arrival of each relocating hare. Winter grounds of resident hares were not delineated. Since six relocating hares died less than 1.5 month after their arrival, the mean number of locations used to delineate winter grounds of relocating hares was 40 ± 9 (median = 45, range: 17–45, n = 19). We extracted percent volume contours (50%, 75%, 95% and 99%) for each seasonal population-level KDEs to represent Arctic hares’ utilization distribution.

All results are expressed as mean ± SD.

## Results

A total of 21 adult females (2 pregnant, 15 lactating, 4 unknown) and four adult males received a satellite collar (Table 1). Body mass of individuals at capture averaged 4.69 ± 0.38 kg for females and 3.90 ± 0.16 kg for males. We obtained 4139 locations (after filtering) with an average of 165 ± 75 locations (range: 33–333) per individual. On average, each hare was monitored for 180 ± 79 days (range: 48–346).

### Fall relocation

Twenty-one hares (19 F, 2 M) moved from their summer to winter grounds (Fig. 1b,c; Supplementary material S1). Summer MCPs of relocating individuals averaged 10.4 ± 13.3 km^2^ (F: 10.3 ± 14.0 km^2^, n = 19; M: 10.7 ± 2.1 km^2^, n = 2) while the MCPs of resident individuals averaged 14.1 ± 4.1 km^2^ (F: 12.8 ± 0.2 km^2^, n = 2; M: 15.4 ± 6.5 km^2^, n = 2). Relocating hares left their summer grounds between 11 August and 17 September (26 August ± 12 days) and arrived on their winter grounds from 10 September to 5 November (6 October ± 13 days). Departures from summer grounds were relatively synchronized, with 80% (17/21 hares) of relocating hares leaving within 25 days (11 Aug–4 Sep). Arrivals on winter grounds were also relatively synchronized with 71% (15/21 hares) arriving within 21 days (22 Sep–12 Oct). Daily hare movement rates differed between the summer residency, fall relocation and winter residency movement phases (F = 66.93, p < 0.001). Before relocation, daily hare movement rates averaged 1.3 ± 0.5 km (n = 1–19) while they increased to 4.3 ± 1.6 km during relocation (n = 15–21, Fig. 3, p < 0.001). A maximum daily average of 11.6 km was observed on day 33 of the relocation period, when seven hares traveled > 15 km. After day 50, hare movement rates decreased, averaging 1.7 ± 0.9 km (n = 1–19), similar to values observed before fall relocation (p = 0.287). The daily movement rate of resident hares during the relocation period averaged 1.2 ± 0.5 km (n = 3–4).

The mean duration of fall relocation was 42 ± 10 days (range: 15–55, n = 21) while the minimum cumulative distance traveled during relocation averaged 198 ± 62 km (range: 113–388, n = 21). Some hares made temporary stopovers while relocating between summer and winter grounds (see e.g. F1, F2, F5 in Supplementary material S1).

### Winter residency

**Figure 4:**
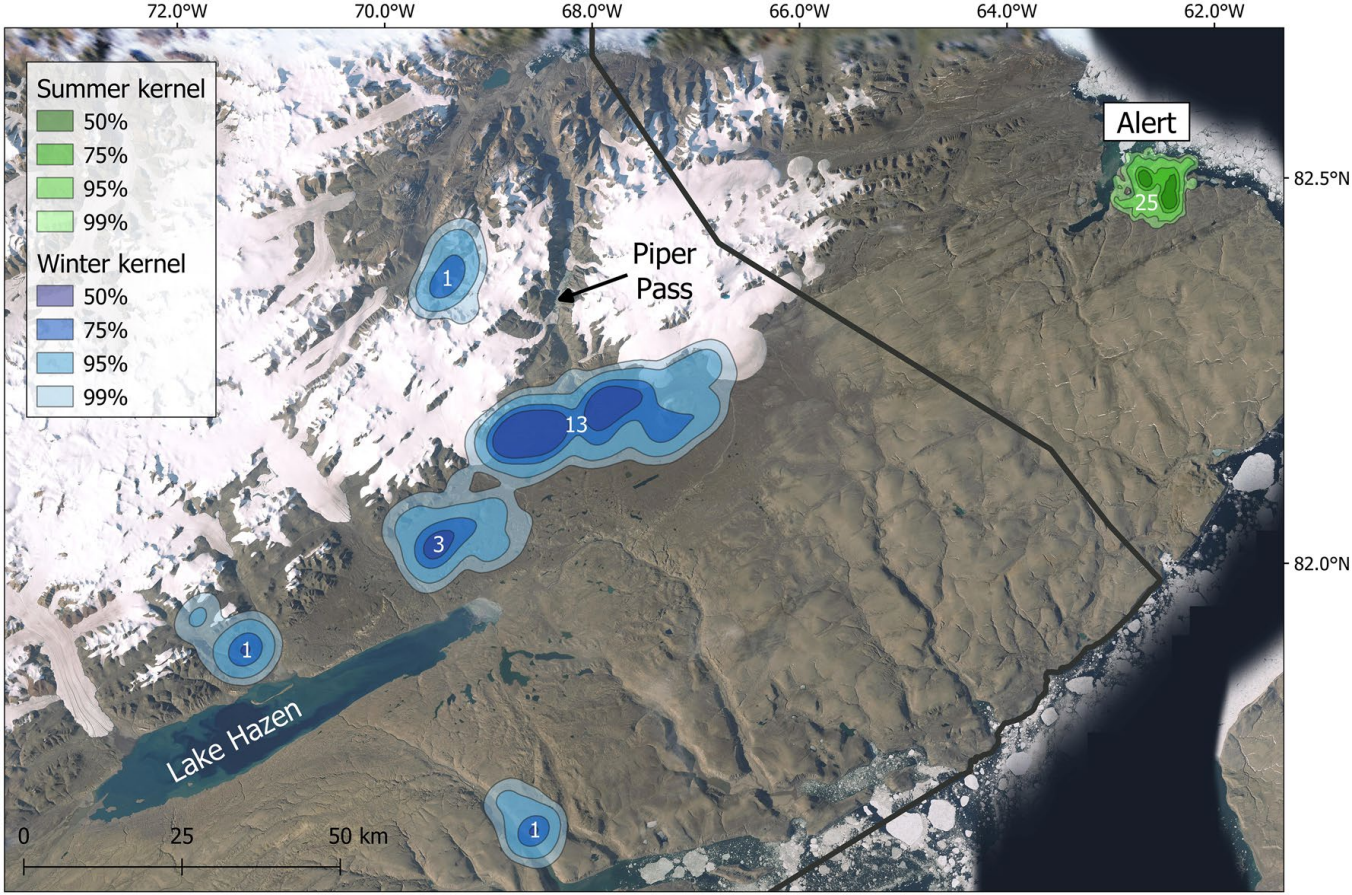
Kernel density distributions of Arctic hares, showing residency areas during summer (green shades, n = 25) and winter (blue shades, n = 19). Kernel density contours at the 50%, 75%, 95% and 99% levels are shown for each season. White numbers indicate the number of individuals present in the smoothed polygons of the 75% kernel density contours. The northeastern boundaries of Quttinirpaaq National Park of Canada are represented by a black line. This map was created using QGIS 3.8.3^40^. Satellite imagery was obtained from: Esri, USGS | Esri, HERE, Garmin, FAO, NOAA, USGS, NRCan, Parks Canada | Earthstar Geographics.

Kernel densities indicated that the main winter grounds of Artic hares captured at Alert were located within the Lake Hazen basin in Quttinirpaaq National Park (Fig. 4). One female initially headed towards Lake Hazen but then turned back and settled close to Alert (F9 in Supplementary material S1). Considering that five hares remained near Alert (including F9), and one died during relocation (F8), the kernel density area for the Lake Hazen basin winter grounds included 19 individuals. The 75% kernel density contour for the Lake Hazen winter grounds covered 283.8 km^2^, which is 12 times larger than the same contour for their summer grounds (23.8 km^2^). Upon arrival, Arctic hares congregated mainly in two areas, one group (68%, n = 13) in a valley at the southern end of Piper Pass (75% kernel density contour = 184.9 km^2^) and another group (16%, n = 3) near the northern tip of Lake Hazen (75% kernel density contour = 42.7 km^2^) (Fig. 4). Only three hares were not spatially connected to these two groups: F16 went further south, F6 settled near the coast, and F17 went to the other side of the ice cap and glaciers (Fig. 4 and Supplementary material S1). Winter MCPs averaged 83.4 ± 118.7 km^2^ (F: 90.9 ± 123.1 km^2^, n = 18; M: 15.5 ± 3.9 km^2^, n = 2). The straight-line distance between summer and winter grounds averaged 98 ± 18 km (range: 72–148 km, n = 19) once F9 was excluded, and 94 ± 26 km (range: 8–148 km, n = 20) if included. Finally, winter grounds were generally located southwest (− 112° ± 0.2°; range: − 92.4° to − 131.0°, n = 19) of summer grounds.

Two collars stopped transmitting while hares were still alive (F12 and M2) while 23 were still functional when hares died. Most mortalities (n = 17) occurred from November to February, with a peak in November (n = 8). Cause of death was unknown. Individual F12 was the last individual to be tracked and she was moving away from her winter grounds, towards the east, when her collar stopped transmitting on 20 May 2020 (Supplementary material S1).

## Discussion

We observed long-distance movements of Arctic hares during fall. Movements were synchronized, directional and resulted in the use of two distant seasonal ranges. Most (84%) of 25 collared hares undertook these movements, suggesting a population-wide phenomenon. No individual was tracked until the next summer, due to winter mortality or collar failure, so we cannot confirm any spring return to summer grounds.

### Migration in Arctic hares

In addition to demonstrating synchronized, directional, and long-distance fall movements, our results provide detailed movement metrics such as speed and timing of relocation. During their fall relocation, the cumulative distance traveled by hares largely exceeded 150 km in most cases, with some individuals traveling > 300 km. These are minimum estimates based on only one location per day. Daily movement rates during relocation were twice as high than during the summer or winter sedentary phases. Following relocation, hares settled for the winter at about 100 km from their summer grounds. All the above contrasts strongly with our current understanding of the spatial ecology of lagomorphs, including Arctic hares. Most lagomorphs are considered sedentary, despite a few accounts of relatively long-distance dispersal in some species like the snowshoe (*Lepus americanus*) and the mountain hare (*Lepus timidus*)^51,52^. However, these dispersals rarely exceed 30 km. Suggestions of long migrations have been made for mountain hares in Russia^53^, but empirical evidence is weak. The only possible migrant lagomorph is the black-tailed jackrabbit (*Lepus californicus*), but observed movements were ≤ 35 km and migration over a full annual cycle was never observed^54^.

Three lines of evidence suggest that the observed movement pattern reflects migratory behavior. First, the fall departure of adults from their summer grounds was both massive and synchronized, as is often observed in migrations^55–57^. We know of no instance of adult dispersal in mammals that is characterized by such frequency of occurrence and such synchronicity. Although several hare species have been tracked, recorded movements do not usually exceed a few tens of kilometers, and adult movements do not occur massively and do not present such synchronicity^58^. Second, migrations are highly directional movements^59^, as observed in this study. It would be surprising to observe such directionality if breeding dispersal was at play, although local geography and topography did constrain the direction of movements. Third, the presence of distinct seasonal ranges used alternatively during the year is the true hallmark of migrations^10,11^ and we did observe that hares relocating from Alert used during the winter a common area in the Lake Hazen basin.

We note that four hares showed range residency at Alert, and one (F9) reversed her relocation path to settle ca. 8 km from her summer grounds, indicating potential within-population variation in migratory tactics^60^. Variation in migration patterns has been observed in other mammalian herbivores, including white-tailed deer (*Odocoileus virginianus*)^44^, caribou (*Rangifer tarandus*)^43^ and moose (*Alces alces*)^61^. Such variability often challenges characterization of movement strategies and movement modes from telemetry data. Considering that long-distance movement behavior was never characterized in Arctic hares and that many movement patterns, including in well-known migratory species, do not perfectly fit conceptualized (modelled) movement strategies^43,62^, we chose not to rely on a published approach such as NSD to separate the residency periods from relocation, and rather used the alternative approach described in Fig. 2. Further research relying on GPS technology allowing a more precise and frequent sampling of individual trajectories may allow the implementation of new methods in the future.

Since we could not observe a complete annual track, we cannot determine fidelity of hares to seasonal grounds. Site fidelity to both seasonal grounds could occur, or it could be limited to only the summer or winter grounds, or fidelity to seasonal grounds may not occur at all (nomadism). Individuals showed correlated fall movements and most of them aggregated in the same winter grounds, so the observed movement pattern could also fit the definition of type II nomadism (sensu Mueller and Fagan^63^). Considering that it is between-year regularity that ultimately determines if a movement pattern is migratory or nomadic^10^, tracking individuals over several years will be necessary to determine the type of movement displayed by Arctic hares reproducing at Alert.

Interestingly, we observed that 92% (23/25) of tracked individuals died during the 11 months that elapsed between the first hare was collared at Alert on 14 June 2019 and the last one was tracked near Lake Hazen on 20 May 2020. All but one of these mortalities occurred on winter grounds rather than on summer grounds or during fall relocation. The longevity of Arctic hares is unknown^23^ and, to our knowledge, annual adult survival was only estimated once (0.78), from extrapolation of daily survival rates and in the most southern part of the species range^28^. High adult mortality rate is not uncommon in the genus *Lepus*. For example, a 99.5% annual adult mortality rate was observed during the declining phase of a cyclic snowshoe hare population at Kluane, Yukon^64^. Observations at Alert that are independent from the current study also suggest a high mortality rate in the studied population. First, visual counts of Arctic hares from observation points at Alert strongly decreased from 2017 to 2019 (D. Berteaux, unpublished data), potentially indicating a population crash. Second, re-sightings of ear-tagged hares were rare during that period; of 28 hares ear-tagged (and not collared) in 2018, only two were re-sighted in 2019. We also note that dramatic density fluctuations of Arctic hares have previously been observed at Eureka, 775 km southwest of our study site^25^. Radio-collars have long been used to track several hare species^26,52,65,66^, including under Arctic climates^67^. Our collars weighed 2–3.1% of hare body mass, well in line with studies of snowshoe hares (< 3%^68^, < 5%^51^), European hares (*Lepus europaeus*) (< 3%^69^, 2.1%^66^), and mountain hares (< 3%^69^, 3%^52^). Therefore, we hypothesize that radio-collaring did not affect survival, but further monitoring of the study population is needed to confirm this.

### Understanding animal movements in the High Arctic

New research avenues emerge from our results and can be broadly split into three categories, that is (1) further characterization of the movement strategy of Arctic hares, (2) understanding of the drivers of Arctic hare movements, and (3) theoretical and practical implications. Describing annual movements of a large sample of individuals in the polar desert is a prerequisite to assess the movement strategy of Arctic hares at their northern distribution limit. Critical questions are: 1—do the fall movements observed in 2019 occur every year, 2—do individuals surviving the winter come back to Alert, 3—what proportion of the population regularly undertakes long-distance movements, 4—do annual movements differ according to age and sex, and 5—to what extent do annual movements influence individual fitness.

Deciphering the drivers of Arctic hare movements in the polar desert will require a good understanding of how external factors (e.g., food, predators, snow conditions, access to social information) interact with the internal state (e.g., body condition, physiology, genetically inherited behavioral traits), the navigation capacity and the motion capacity of individuals^70^. A first step involves testing hypotheses about how the distribution of resources in the spatiotemporal landscape correlates with observed movements^63,71^. We hypothesize that hares overwintering in the Lake Hazen basin can access better food conditions and more favorable microclimates than those not doing so. Indeed, the geographical configuration of the terrain around Lake Hazen makes it a polar thermal oasis with milder temperatures during winter, a longer frost-free period and a higher plant productivity than the surrounding region^72^. The above, however, would not explain why individuals spend the summer at Alert. Given that most captured females were pregnant or lactating, a complementary hypothesis is therefore that Alert provides a refuge against predation for females raising leverets. Preliminary evidence suggests that Arctic wolves may be more abundant in Quttinirpaaq National Park than at Alert. Indeed, high concentrations of muskoxen have been reported on the Lake Hazen-Alert Plateau, a low-lying plateau extending north from Lake Hazen^73^. Arctic wolves rely heavily on muskoxen in the polar desert^74^ and it is often assumed that high muskoxen numbers support higher wolf numbers^75^. Arctic hares may be moving to poorer foraging areas during the breeding season as a predator-avoidance strategy, as seen in ungulates such as moose and caribou^76,77^.

The theoretical and practical implications of our results are many. Given the important role of Arctic hares in the polar desert, both as prey^25^ and herbivores^78^, and their large numbers at high latitudes^23^, Arctic hare movements have the potential to impact deeply the seasonal dynamics of local ecosystems. Herds of 100–300 hares are routinely reported in the literature^23^, groups of 1000 individuals have been repeatedly observed on Ellesmere Island^79^, and a biologist referred to the “moving hillside” phenomenon (R.I.G. Morrison, pers. com.) after observing large groups of hares running across slopes in our study area. A large population of moving herbivores could strongly affect plant communities, other herbivores, and predators. Given the above ecological implications, resolving Arctic hare movements would be a major addition to our understanding of regional ecosystems.

A unified understanding of why animals migrate necessitates comparative studies across taxa while accounting for phylogeny^22^. This requires data spanning multiple taxa, whereas published information is, in mammals, severely biased toward Cetacea and Artiodactyla^22^. If confirmed, migration in Arctic hares would thus indicate development of long-distance migration in the order Lagomorpha, a useful addition to the growing database of migrating mammals. A practical implication of our work involves biodiversity conservation at the very northern margin of the Americas. Hares crossed the boundaries of Quttinirpaaq National Park during their relocation. While the Lake Hazen basin constitutes an area with a high degree of protection^80^, it is also important to recognize that movements extend beyond park boundaries and may necessitate an integrative conservation strategy. Finally, the increasing pressure of climate change on Arctic ecosystems^32^ may modify not only the environmental cues used to trigger movements (e.g., spring snow melt or fall snow establishment), but also the spatiotemporal variability of vegetation growth and the extent of the snow-free season, potentially leading to altered movements or a loss of migratory behavior^81,82^.

## Conclusion

We provide the first evidence that Arctic hares are capable of seasonal long-distance movement. Individuals can routinely cover distances exceeding by far any previously reported in the order lagomorph. This new knowledge opens avenues for future research in several fields ranging from animal behavior to ecosystem ecology and conservation biology.

## Supplementary Information

Supplementary Figure S1.
