# Supplementary material for: Long-distance, synchronized and directional fall movements suggest migration in Arctic hares on Ellesmere Island (Canada)
Source: Sci Rep. 2022 Mar 23;12:5003. doi: 10.1038/s41598-022-08347-1 (PMC8943133; doi:10.1038/s41598-022-08347-1)

Supplementary information for

**Long-distance, synchronized and directional fall movements suggest migration in Arctic hares on Ellesmere Island (Canada)**

Jacob Caron-Carrier, Sandra Lai, François Vézina, Andrew Tam, Dominique Berteaux

**Table of contents**

[Supplementary figure S1. Representation of the movements of the 25 Arctic hares tracked during the study. 2](#_Toc97823046)

Supplementary figure S1. Representation of the movements of the 25 Arctic hares tracked during the study. Locations were obtained by Argos telemetry between 15 June 2019 – 31 May 2020 and are represented by green circles (summer locations), orange circles (fall relocation), and blue circles (winter locations). Summer range (green), relocation path (orange dots and line), and winter range (blue) are also presented for relocating individuals. For residents, we present the annual range and its locations (both in purple). The northeastern boundaries of Quttinirpaaq National Park of Canada are represented by a black line. We also present variation through time of the net squared displacement (NSD) for each hare. The NSD is the squared straight-line distance between each location and an initial point. NSD data points are represented by blue dots and the black line is a five-days moving average. NSD is plotted for each hare from its first day of tracking in 2019 to its last day of tracking in 2019 or 2020, depending on individuals. The label in the top right corner of each map gives hare ID (see Table 1). These maps were created using QGIS 3.8.3 (QGIS Development Team, 2021). Satellite imagery was obtained from: Esri, USGS | Esri, HERE, Garmin, FAO, NOAA, USGS, NRCan, Parks Canada | Earthstar Geographics.


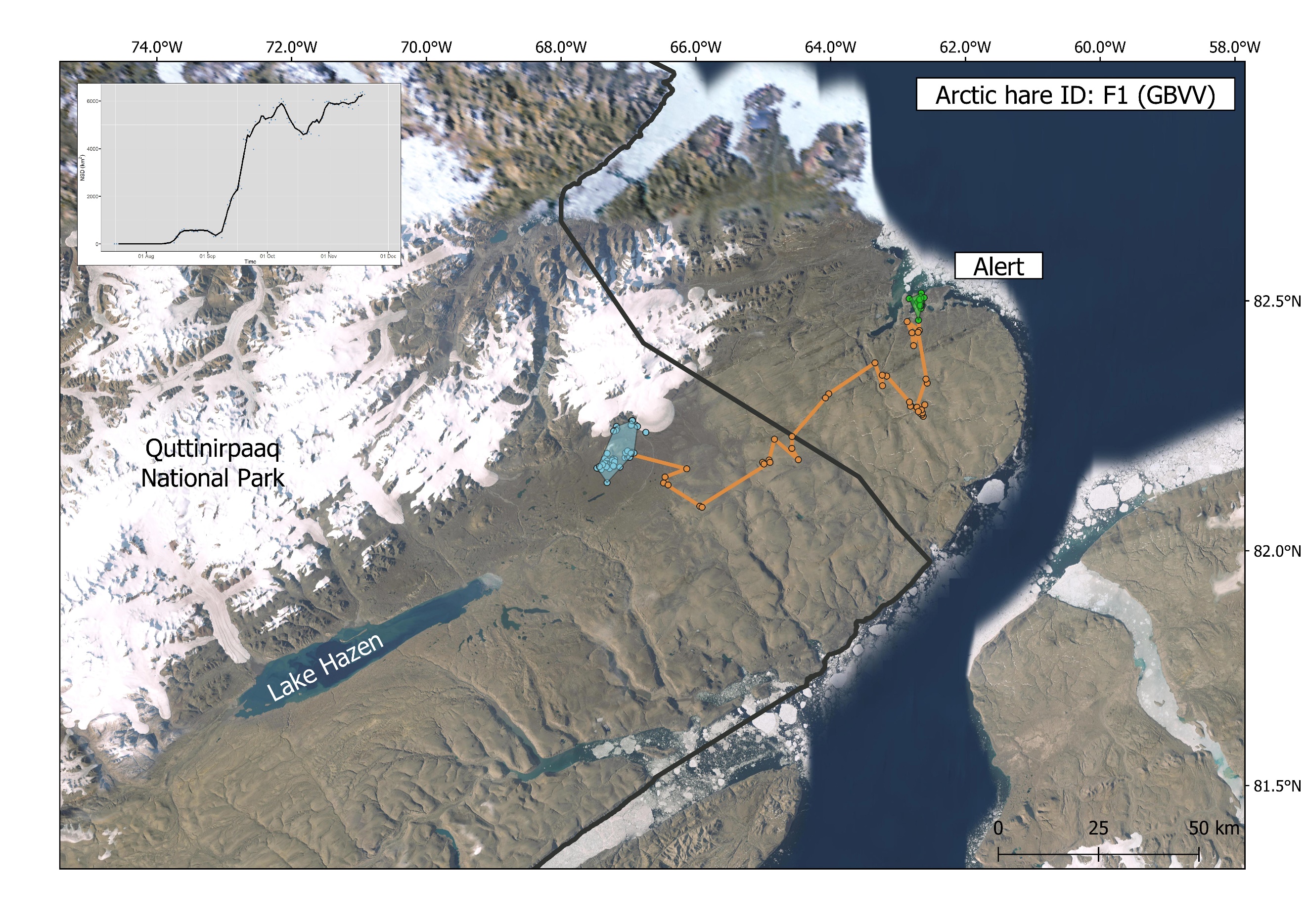


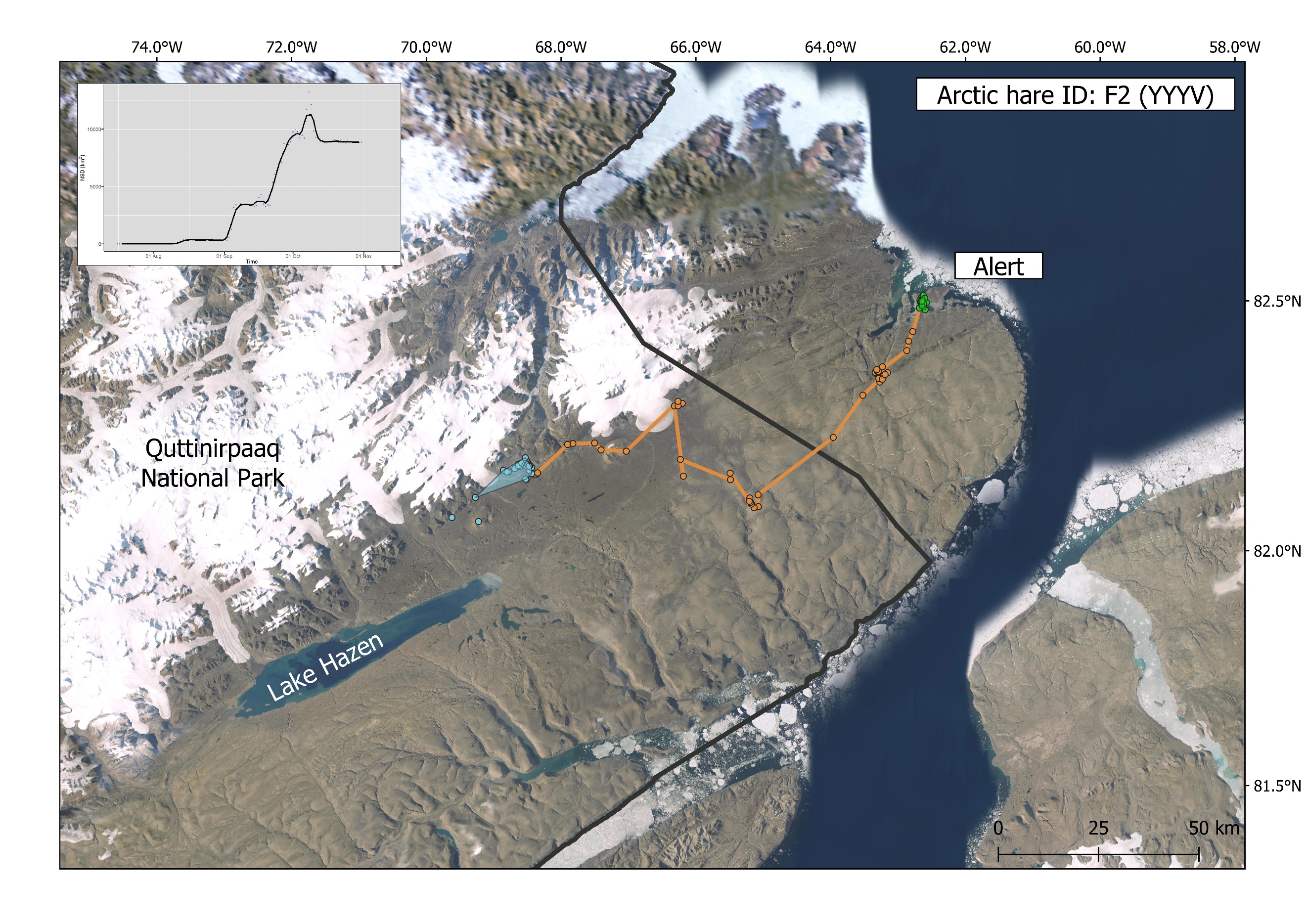


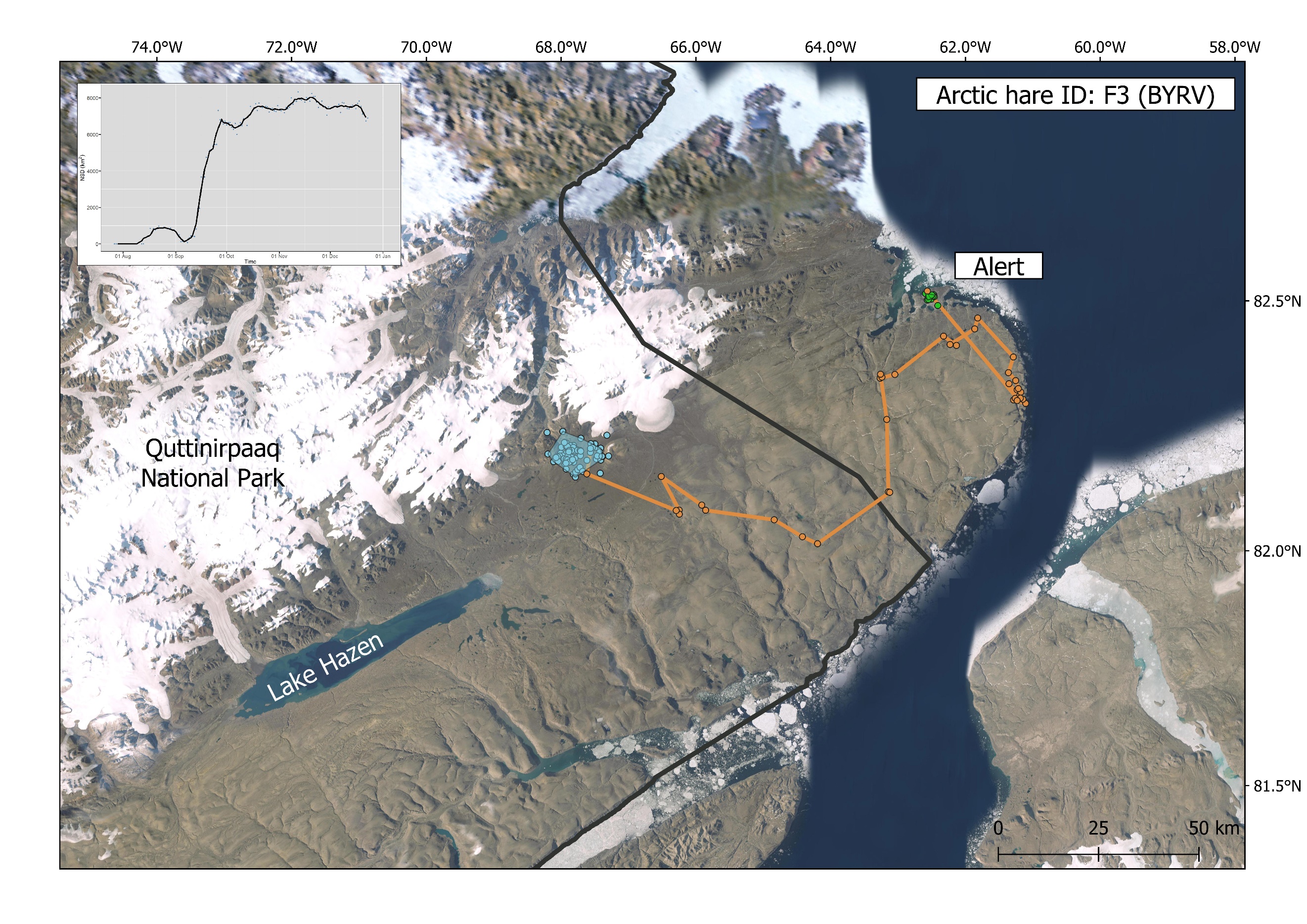


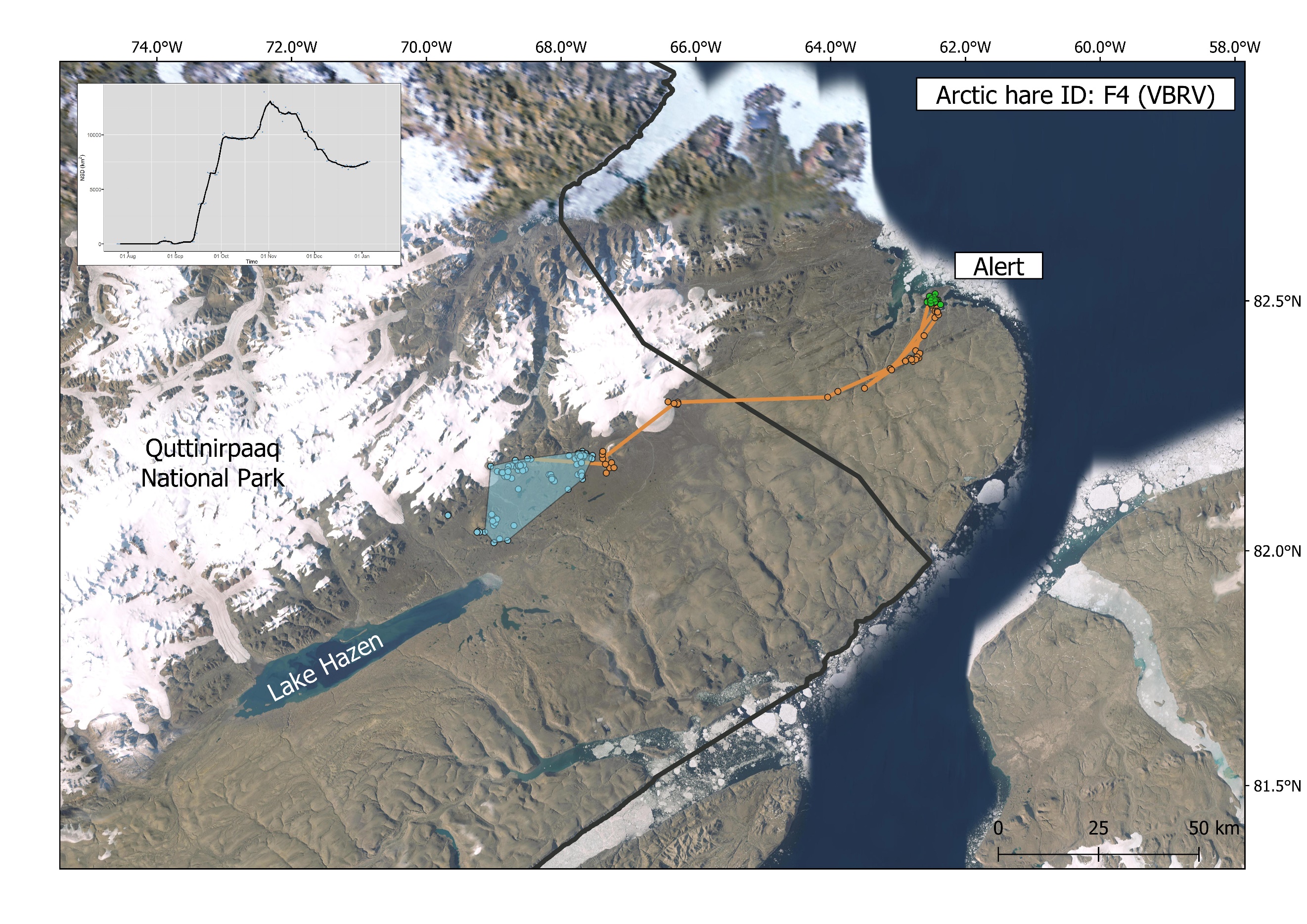


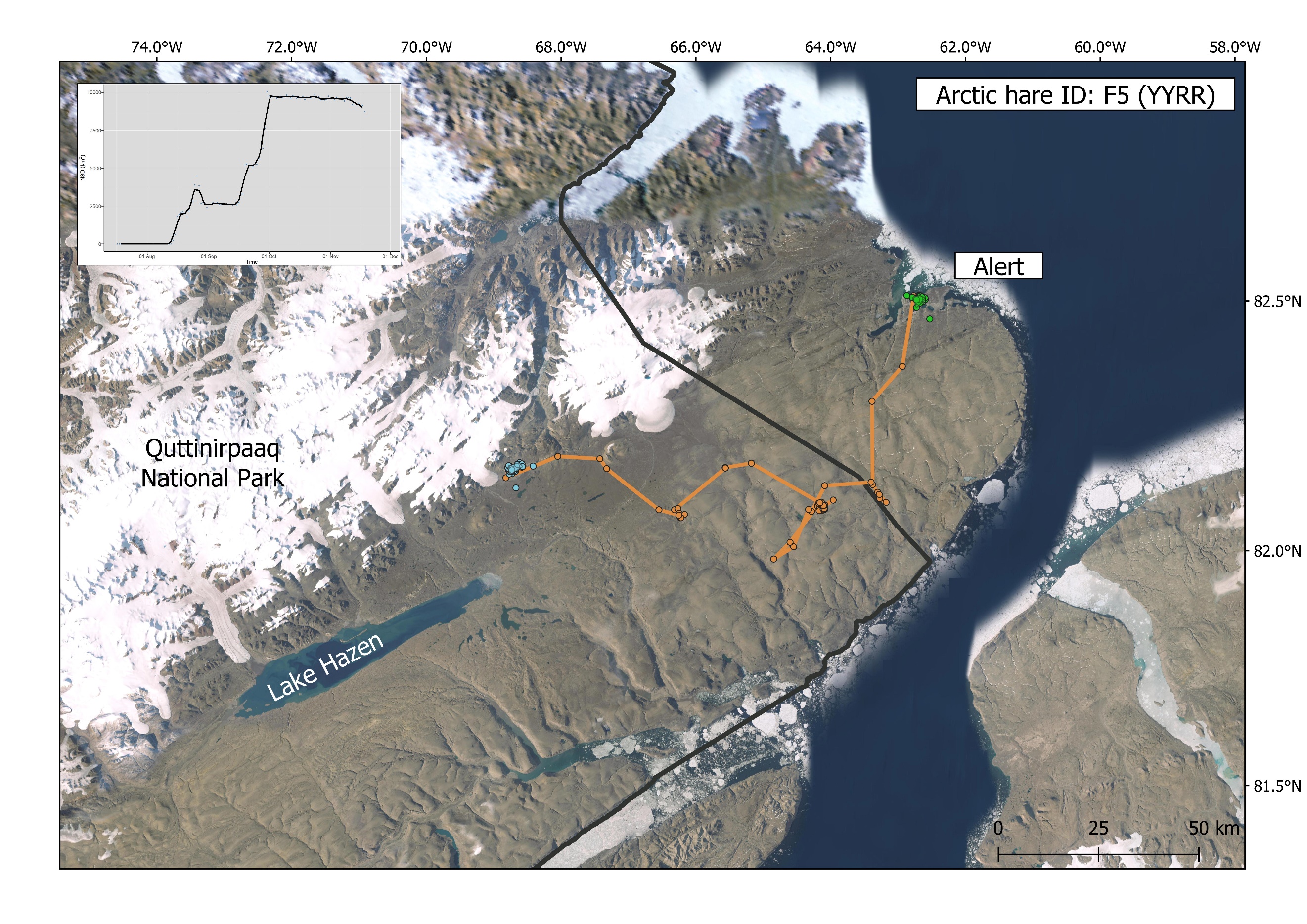


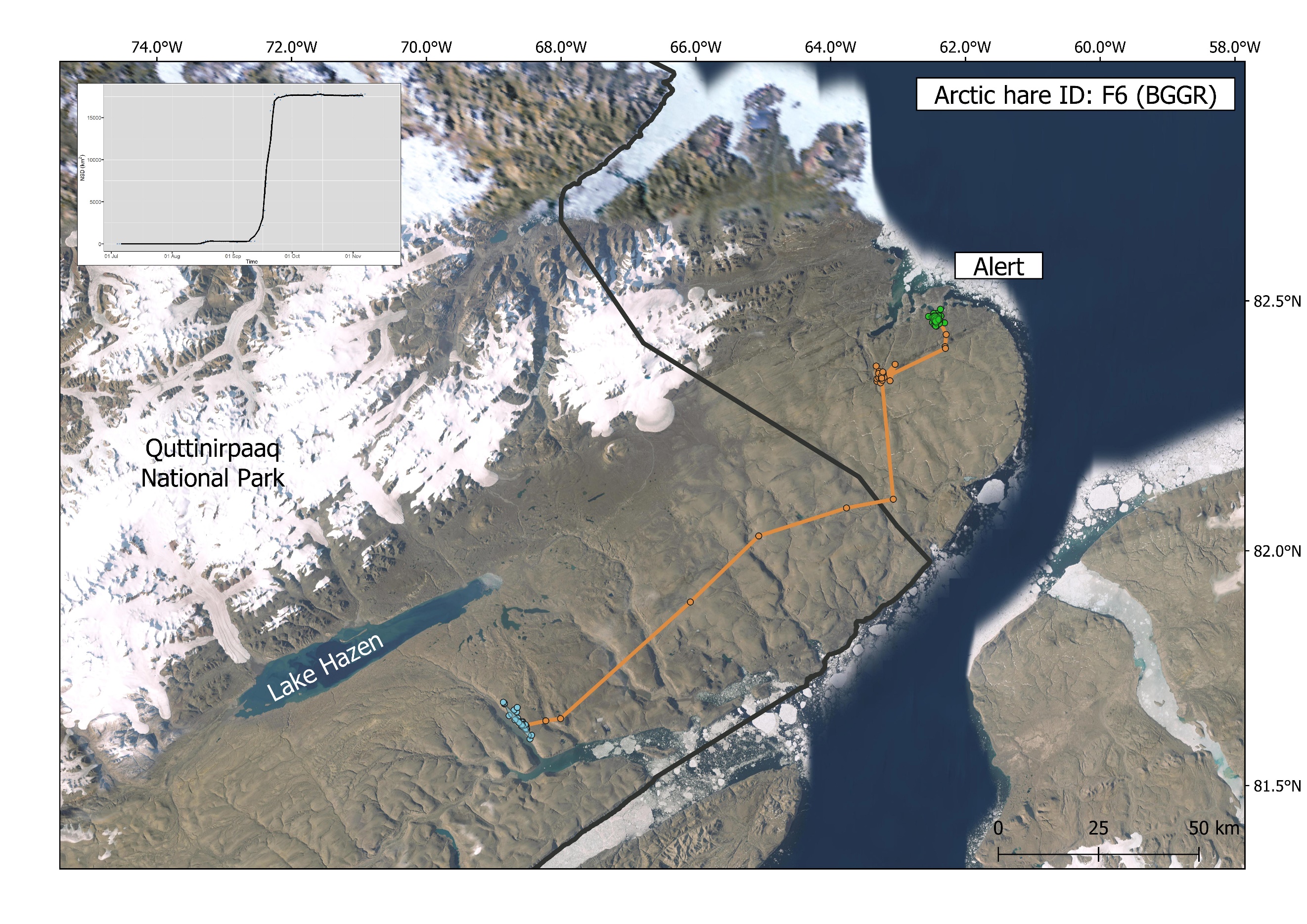


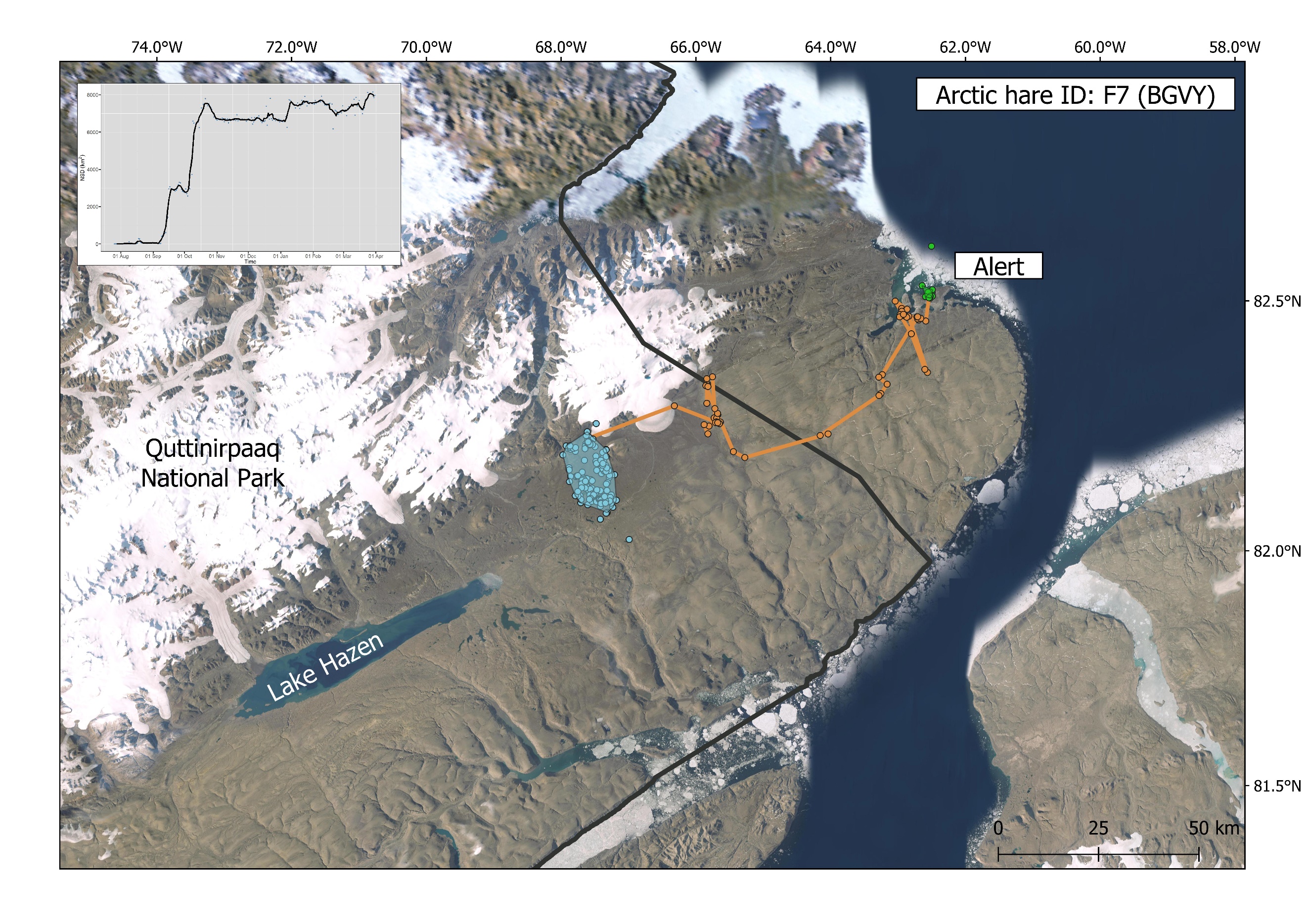


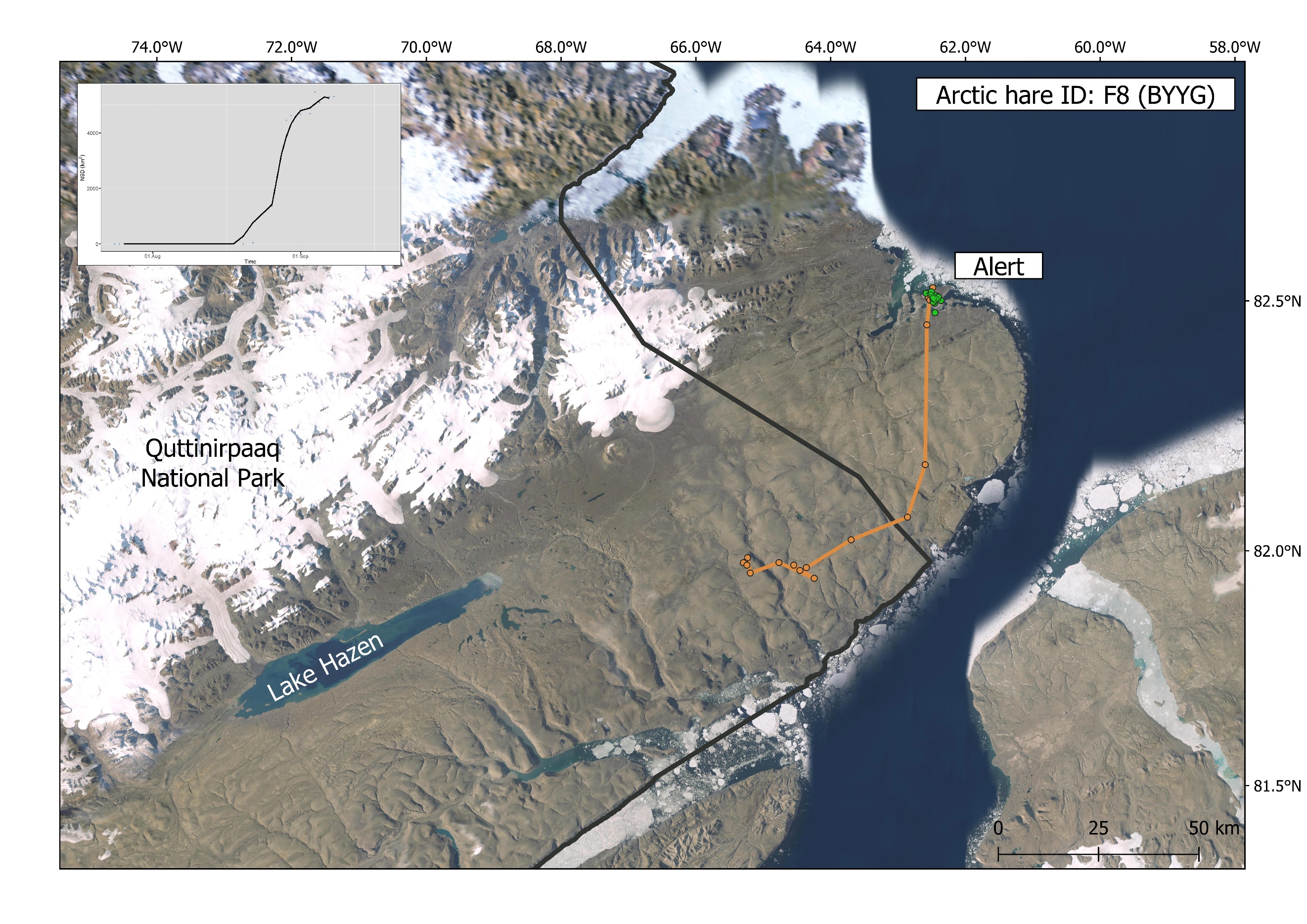


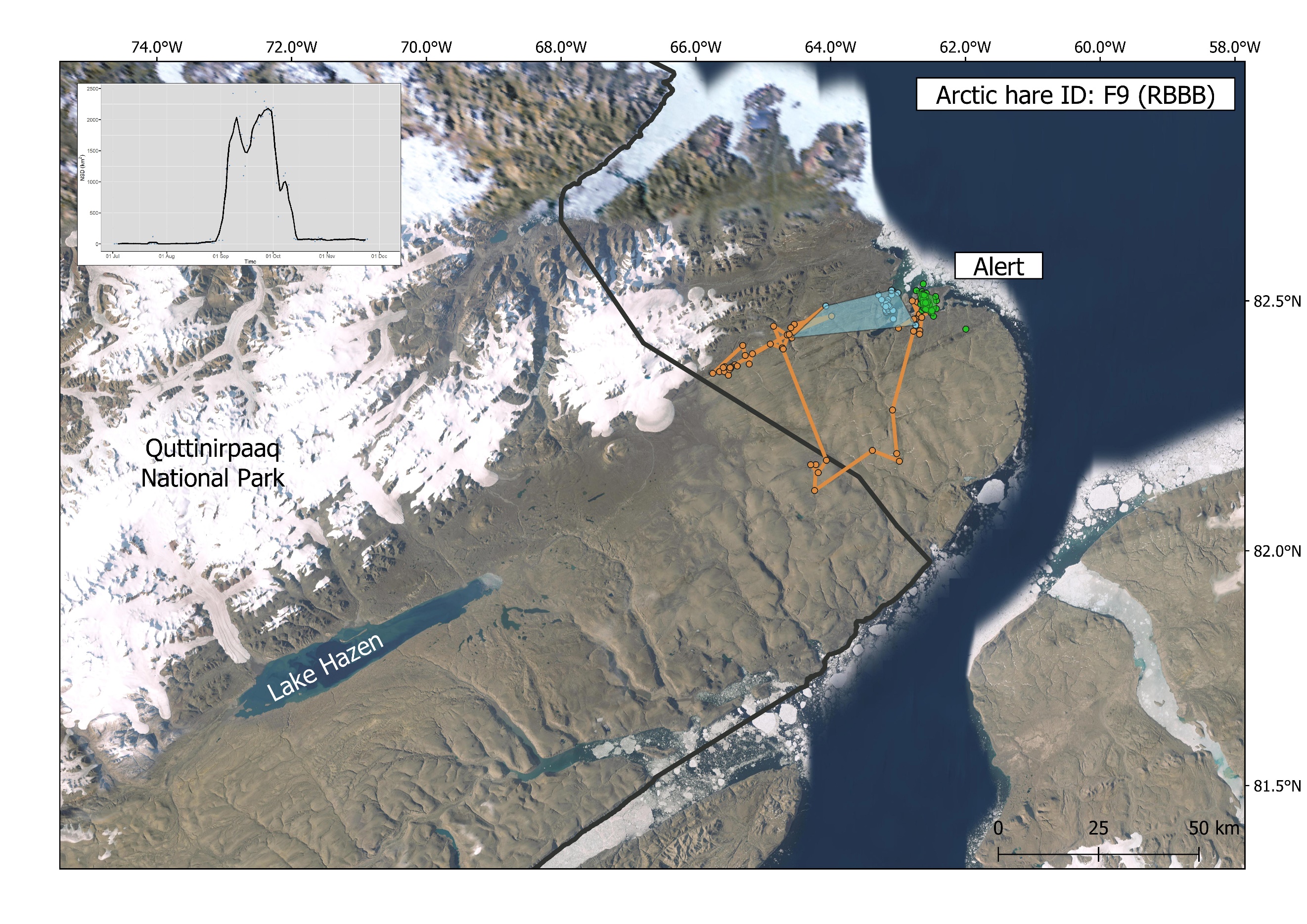


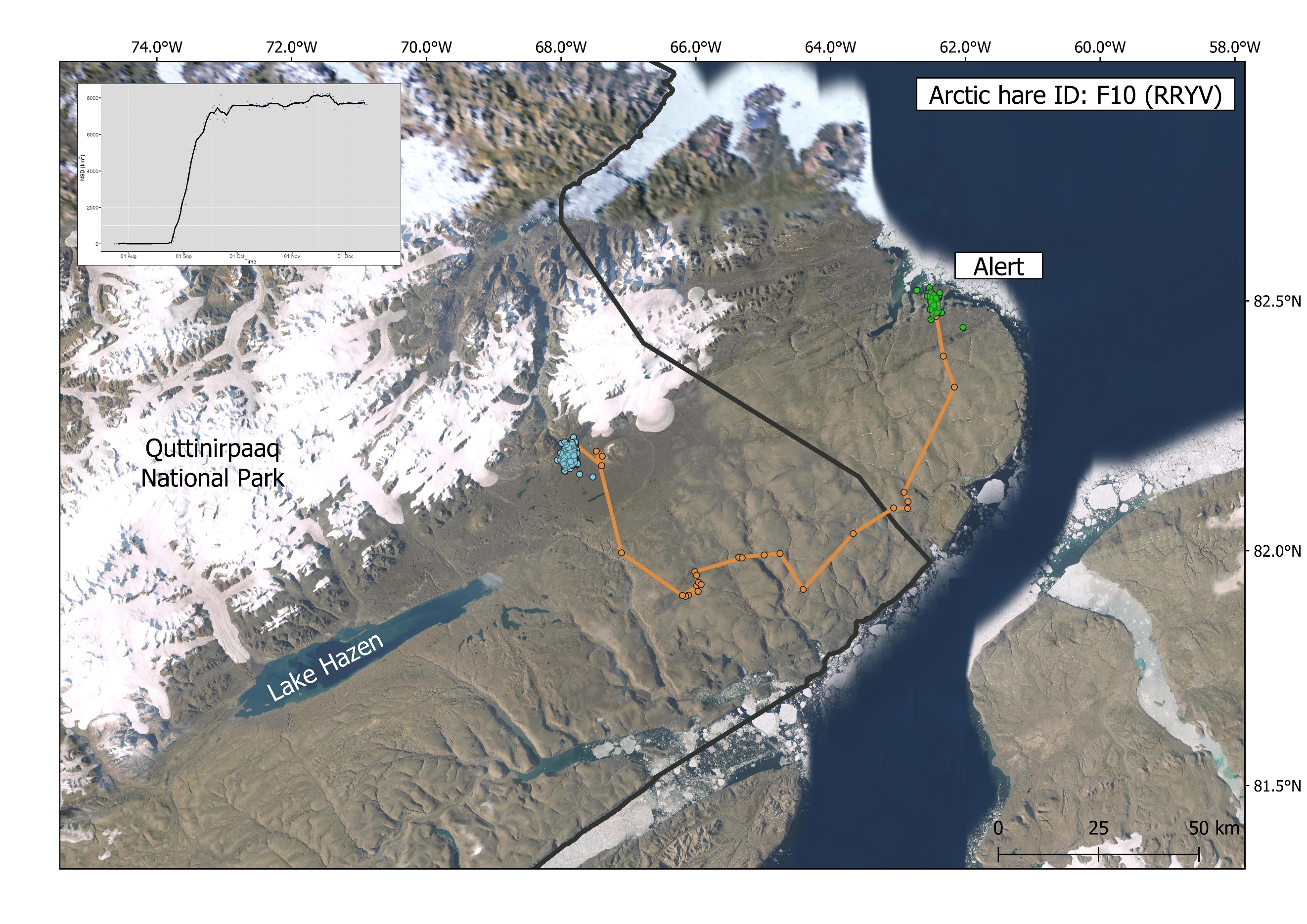


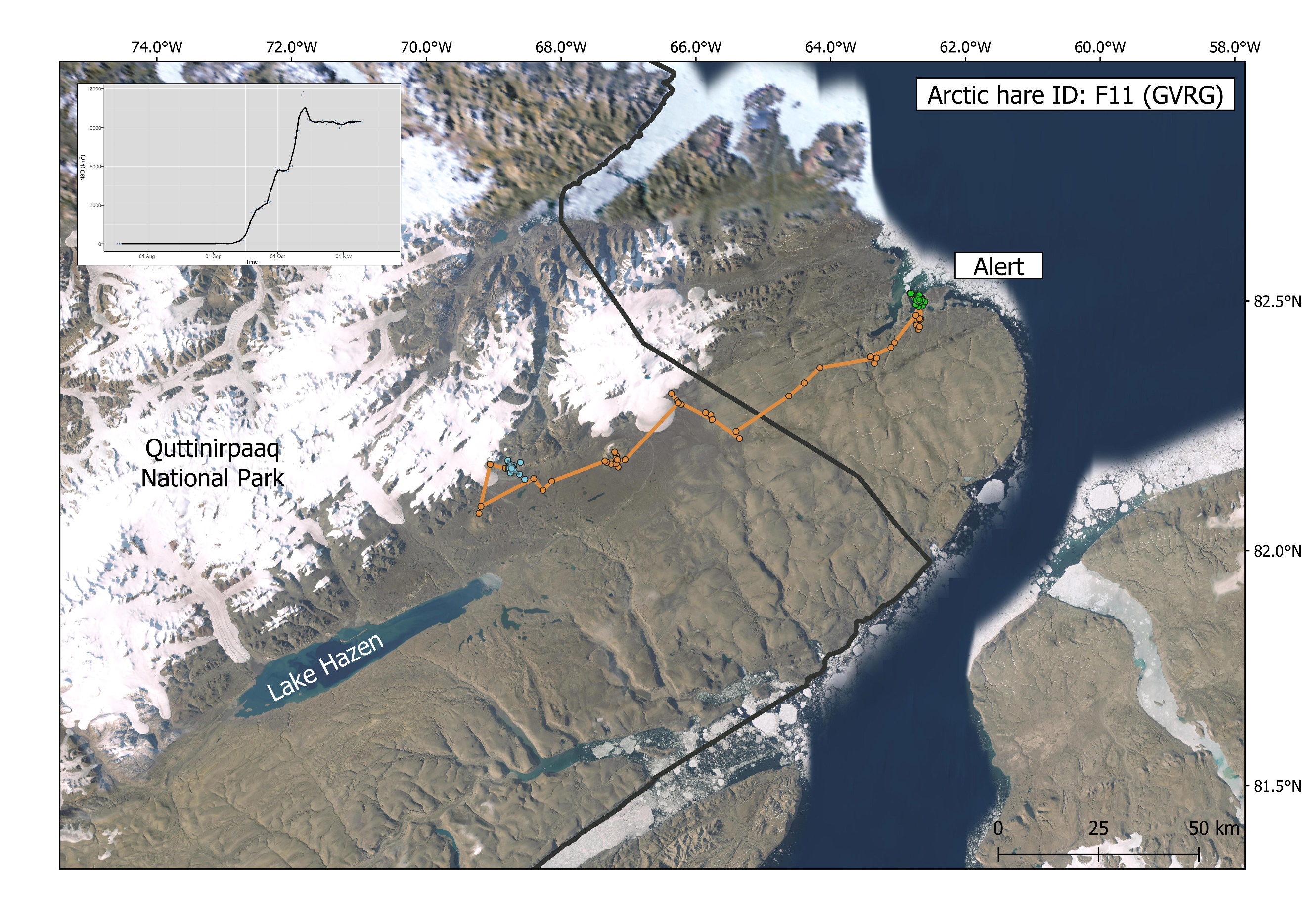


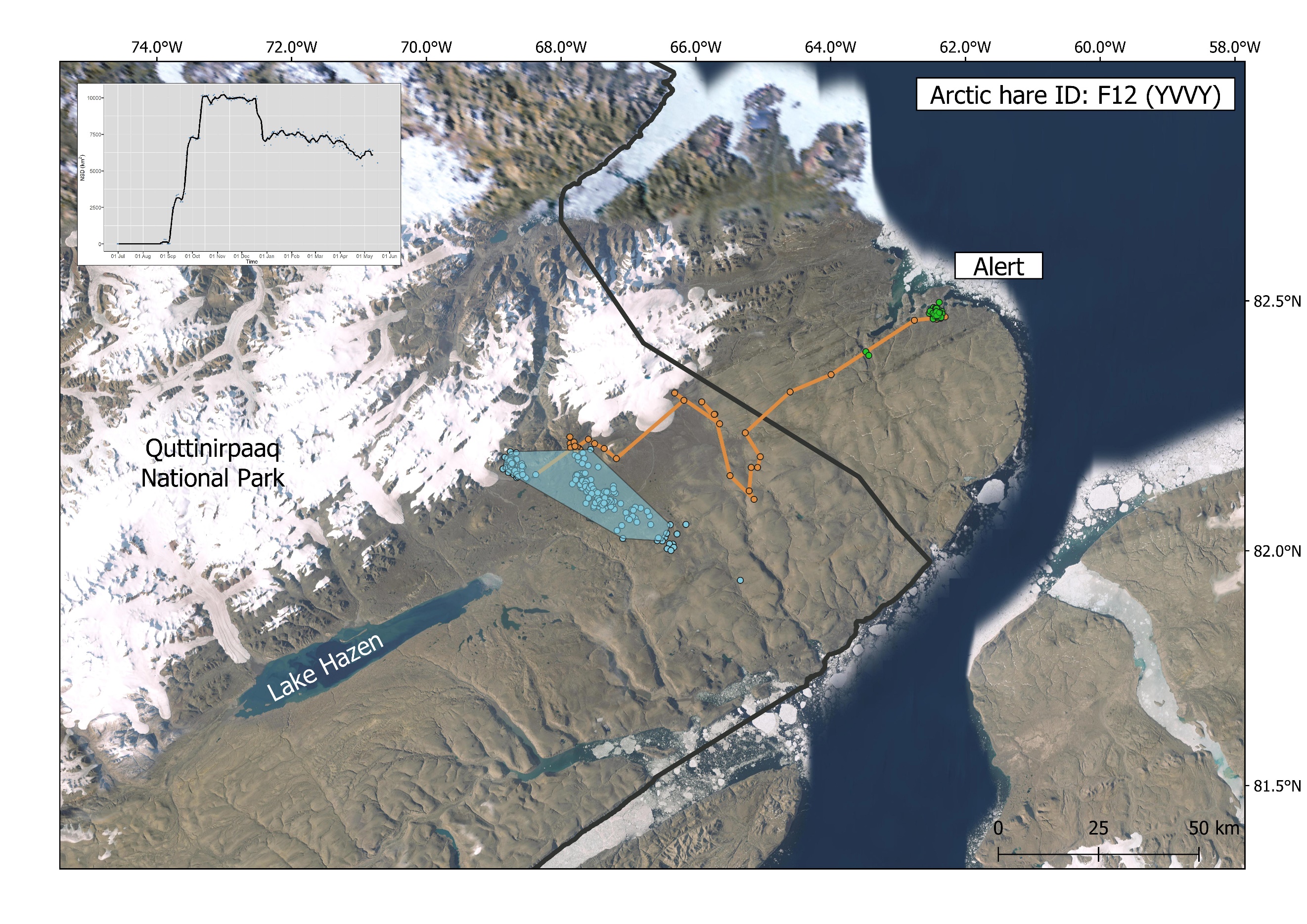


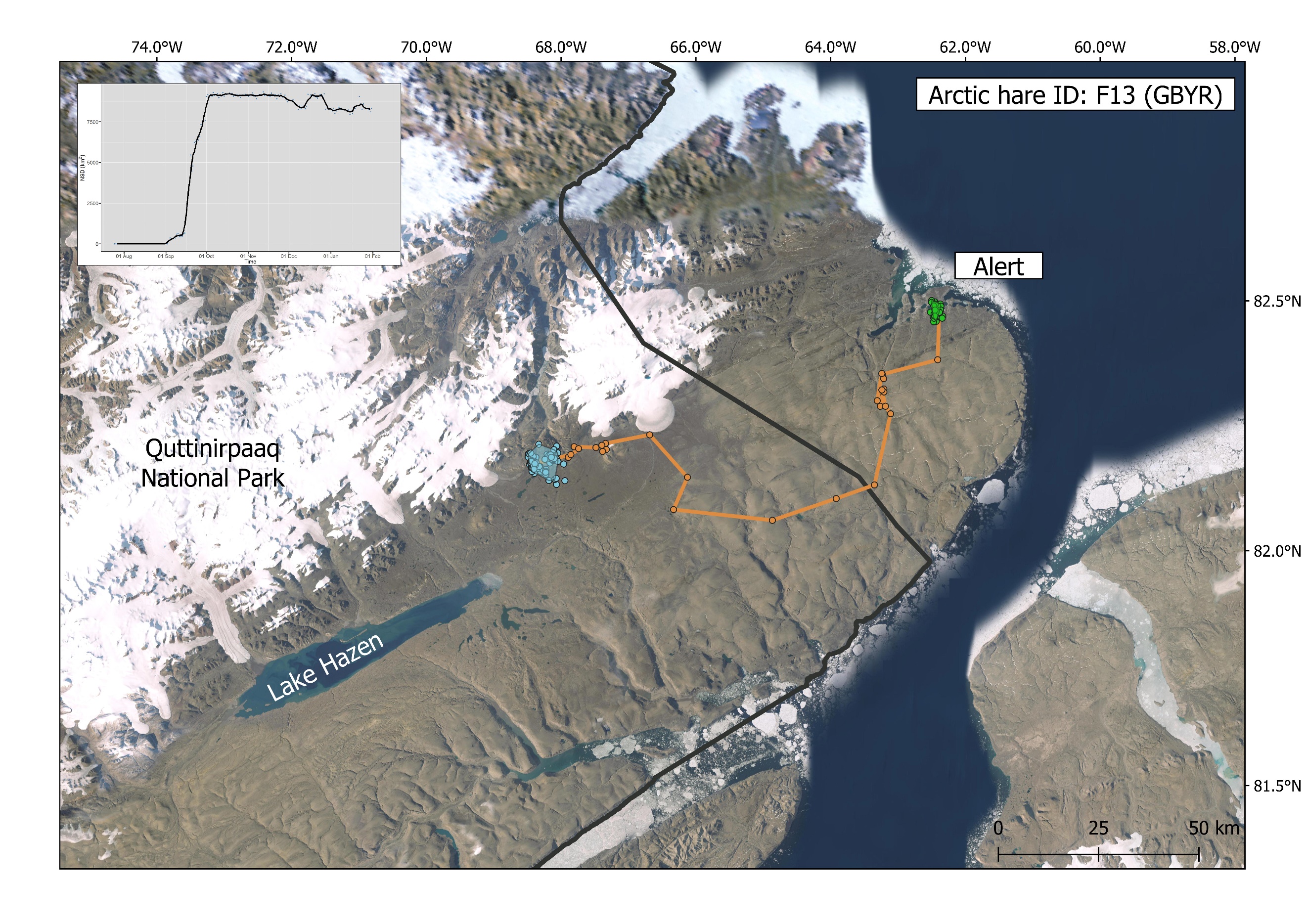


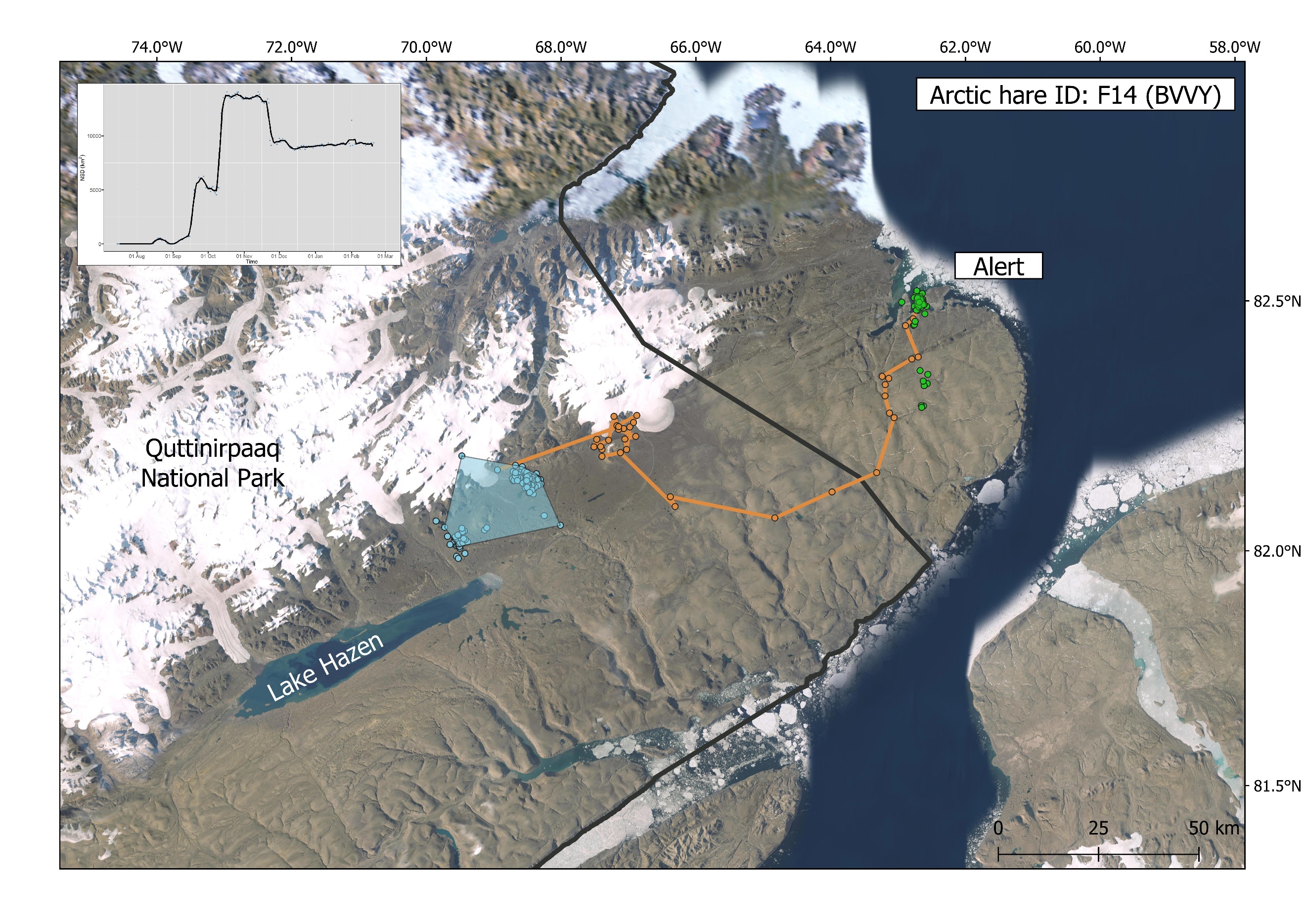


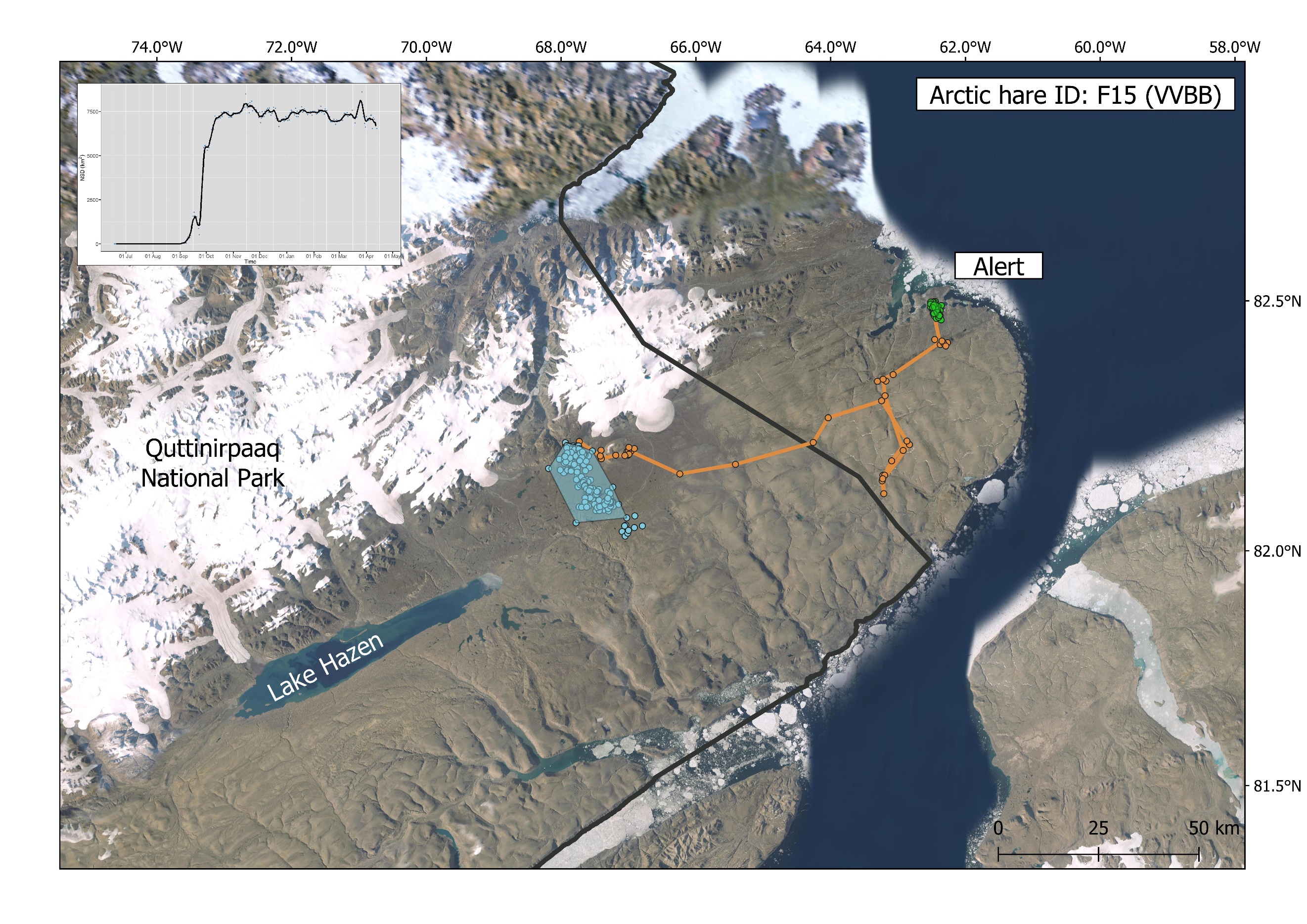


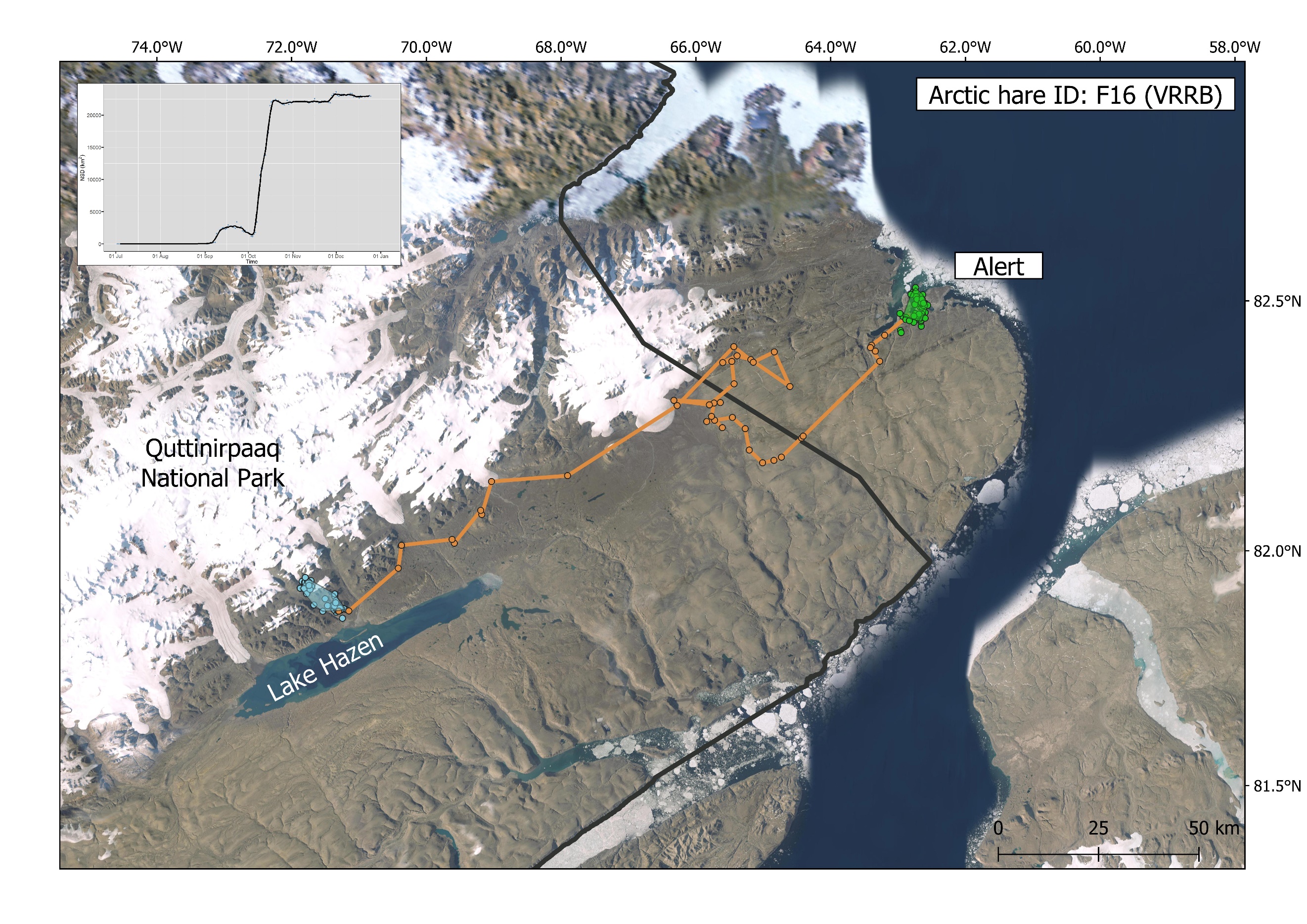


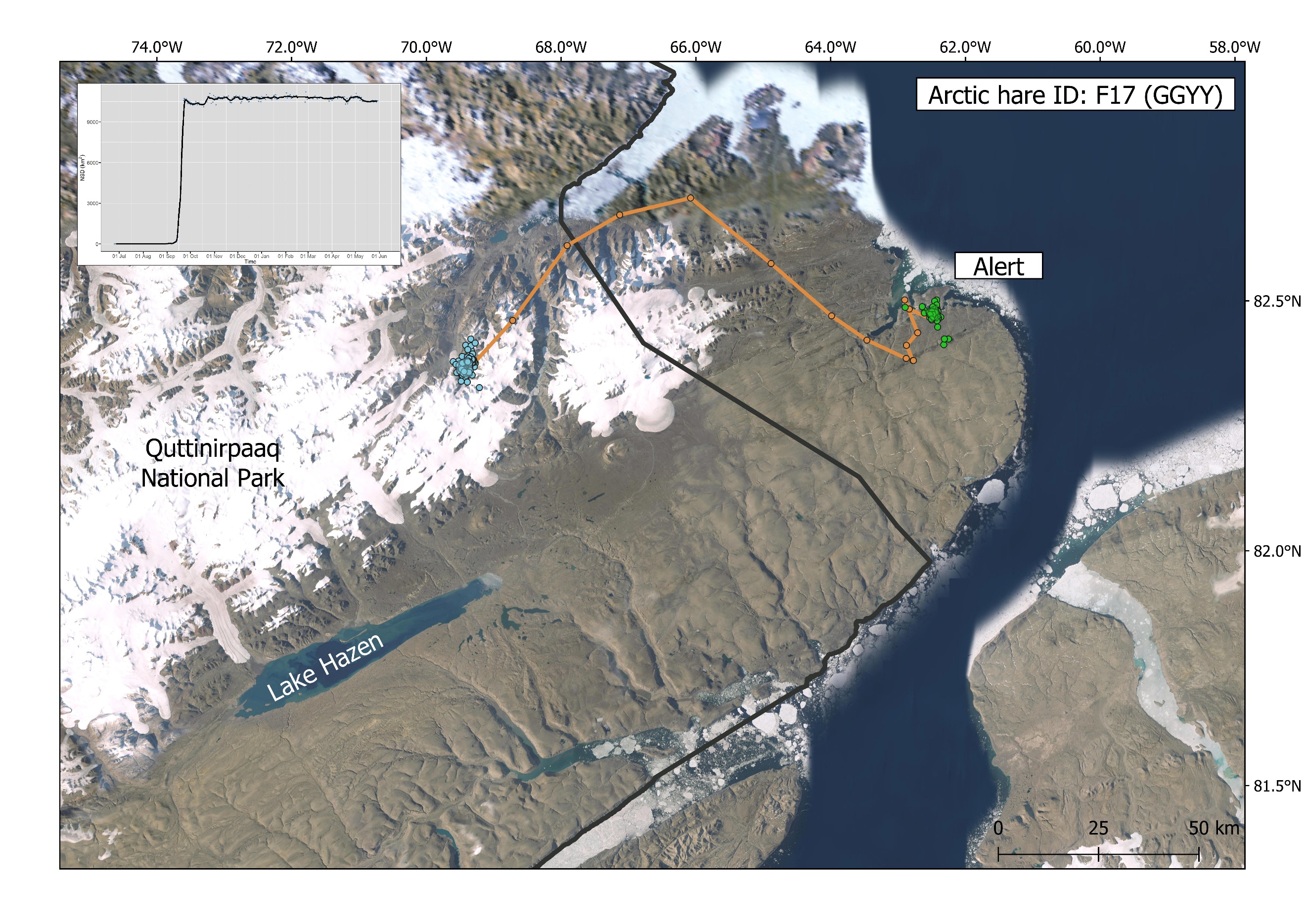


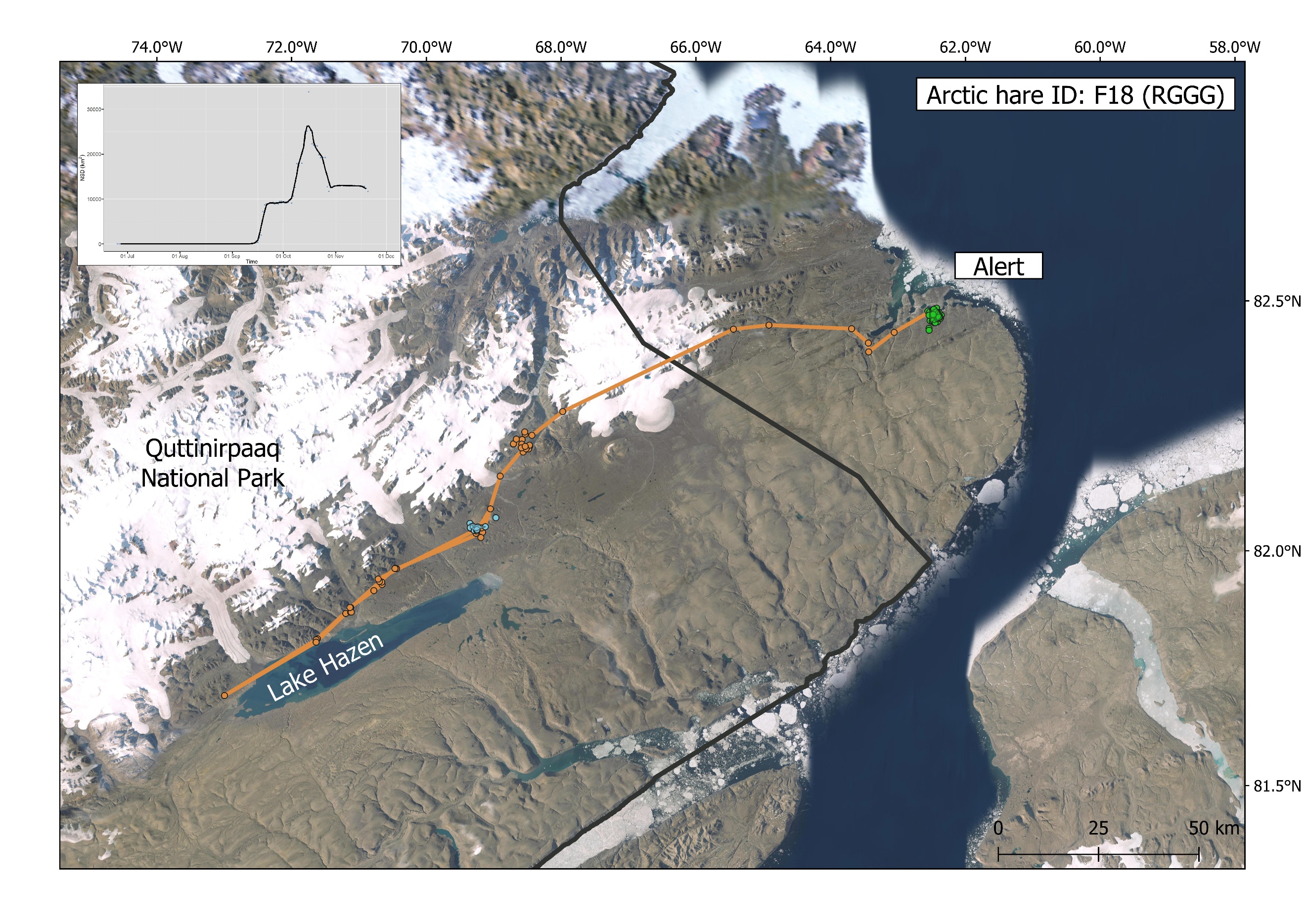


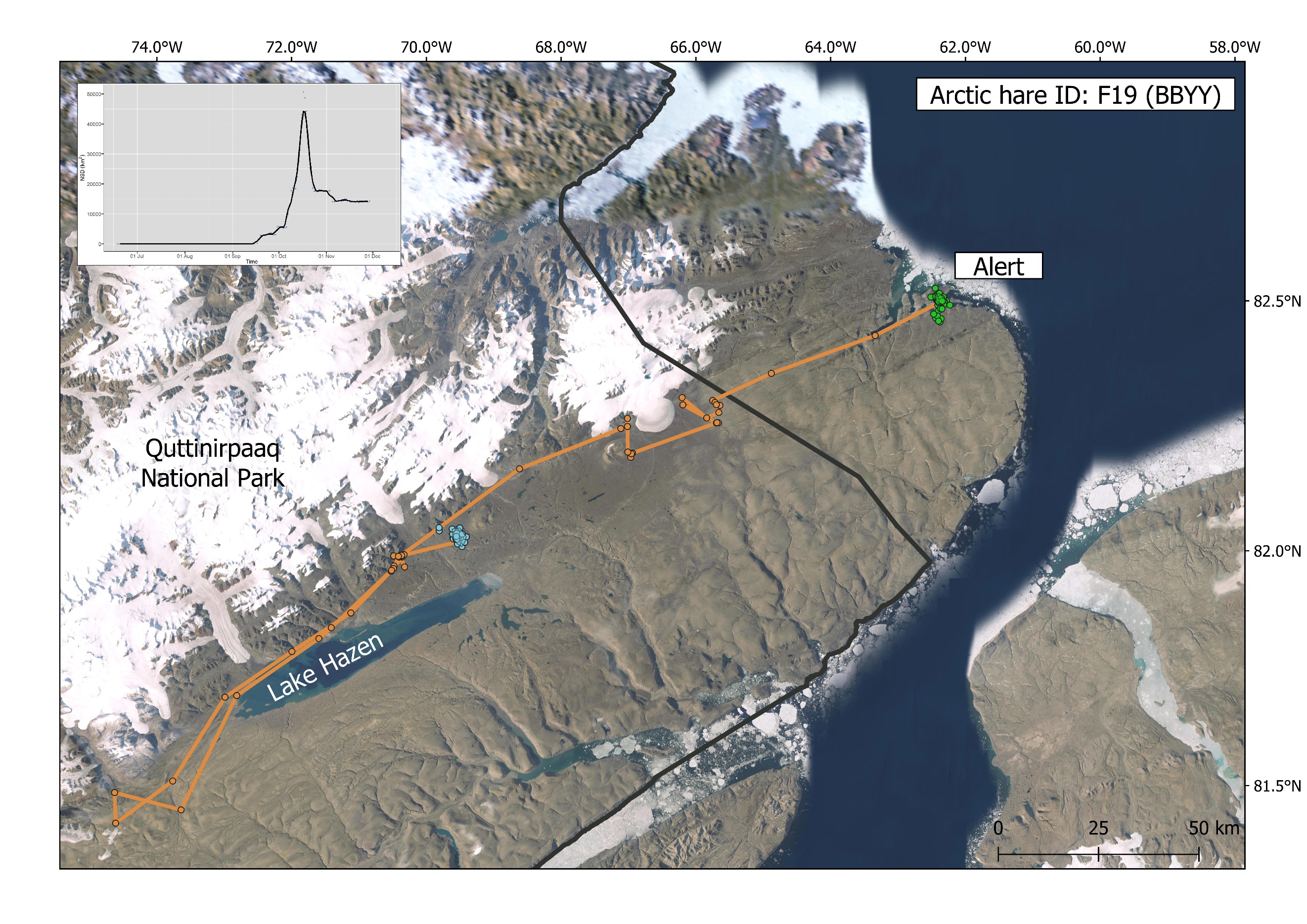


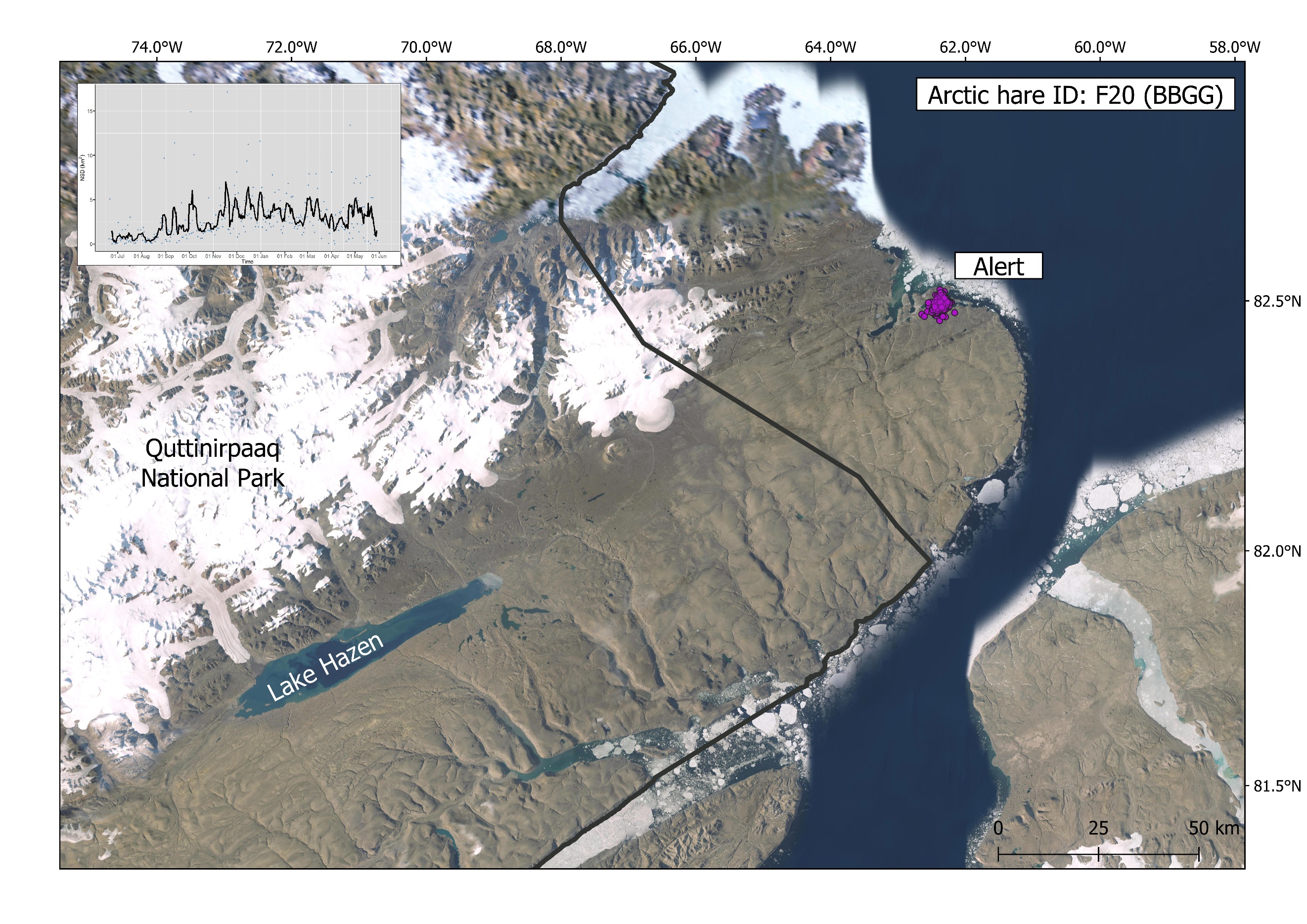


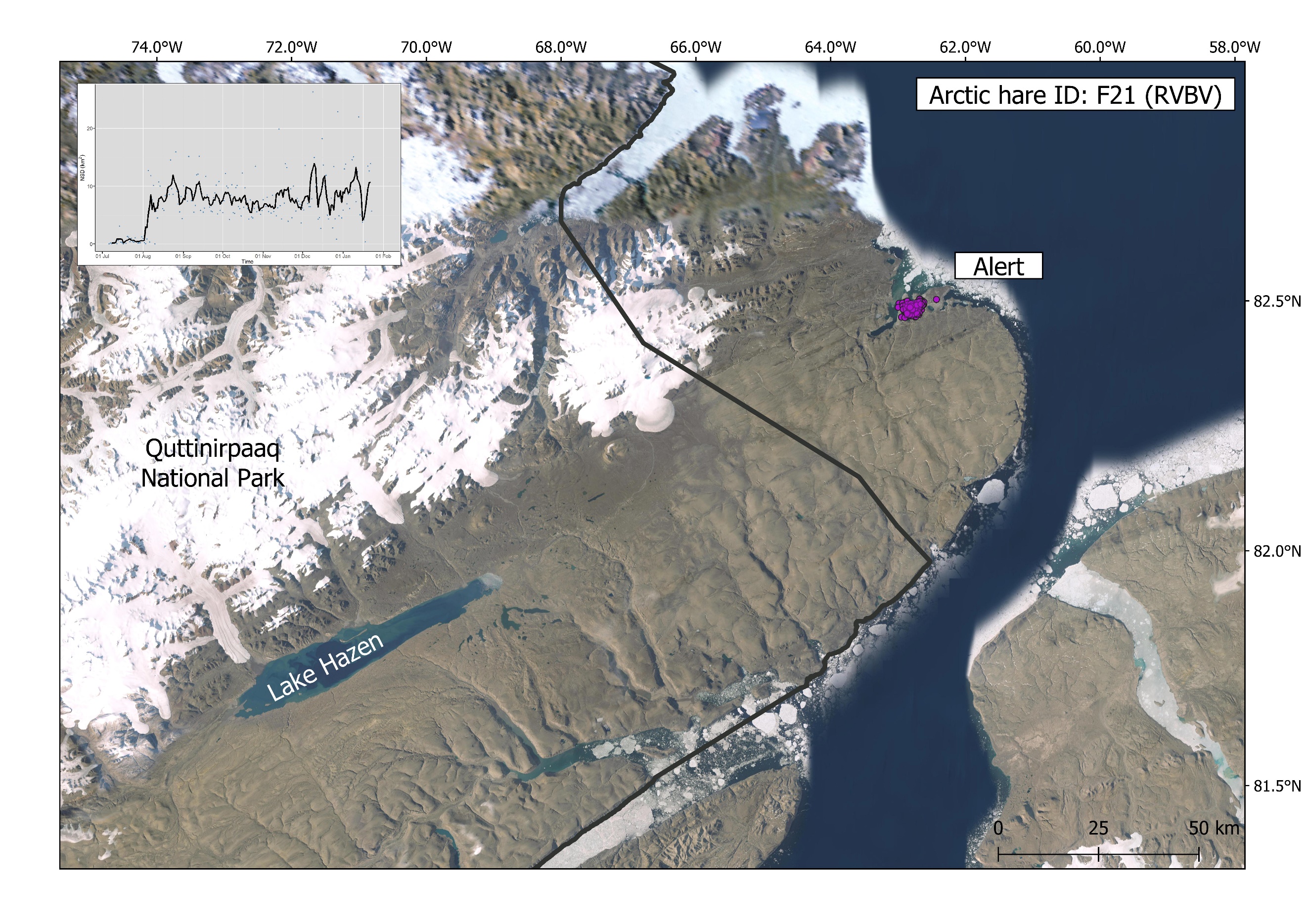


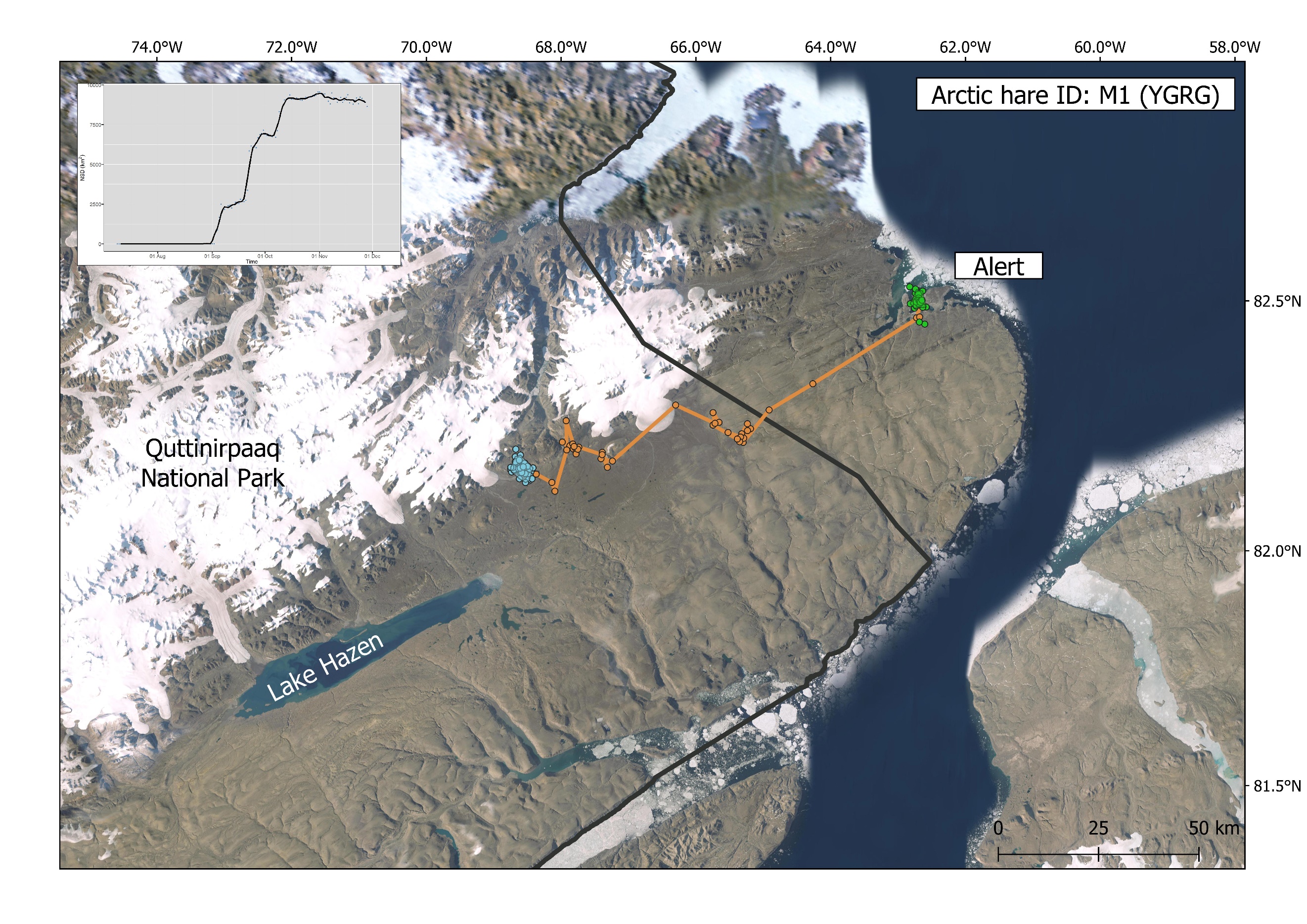


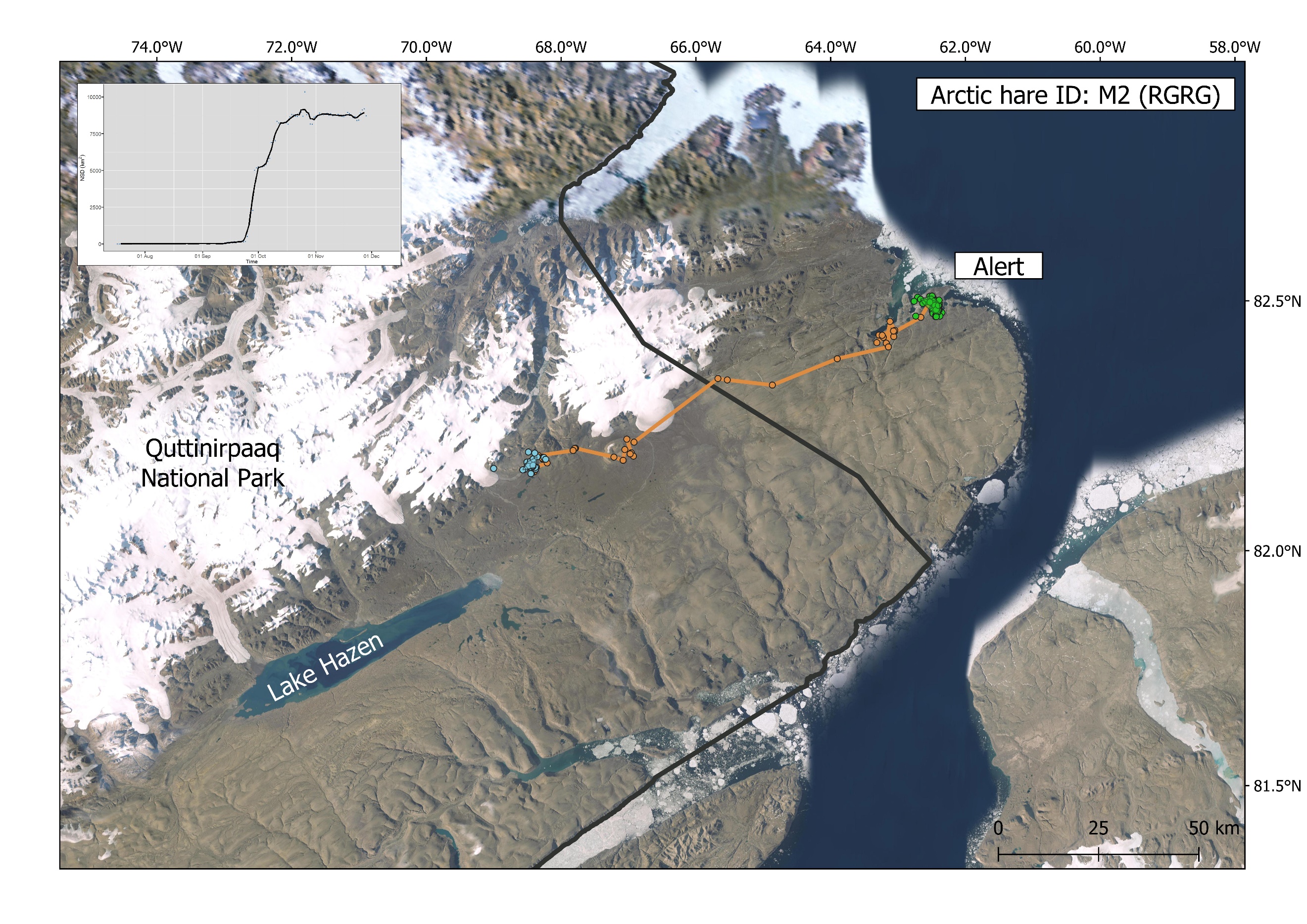


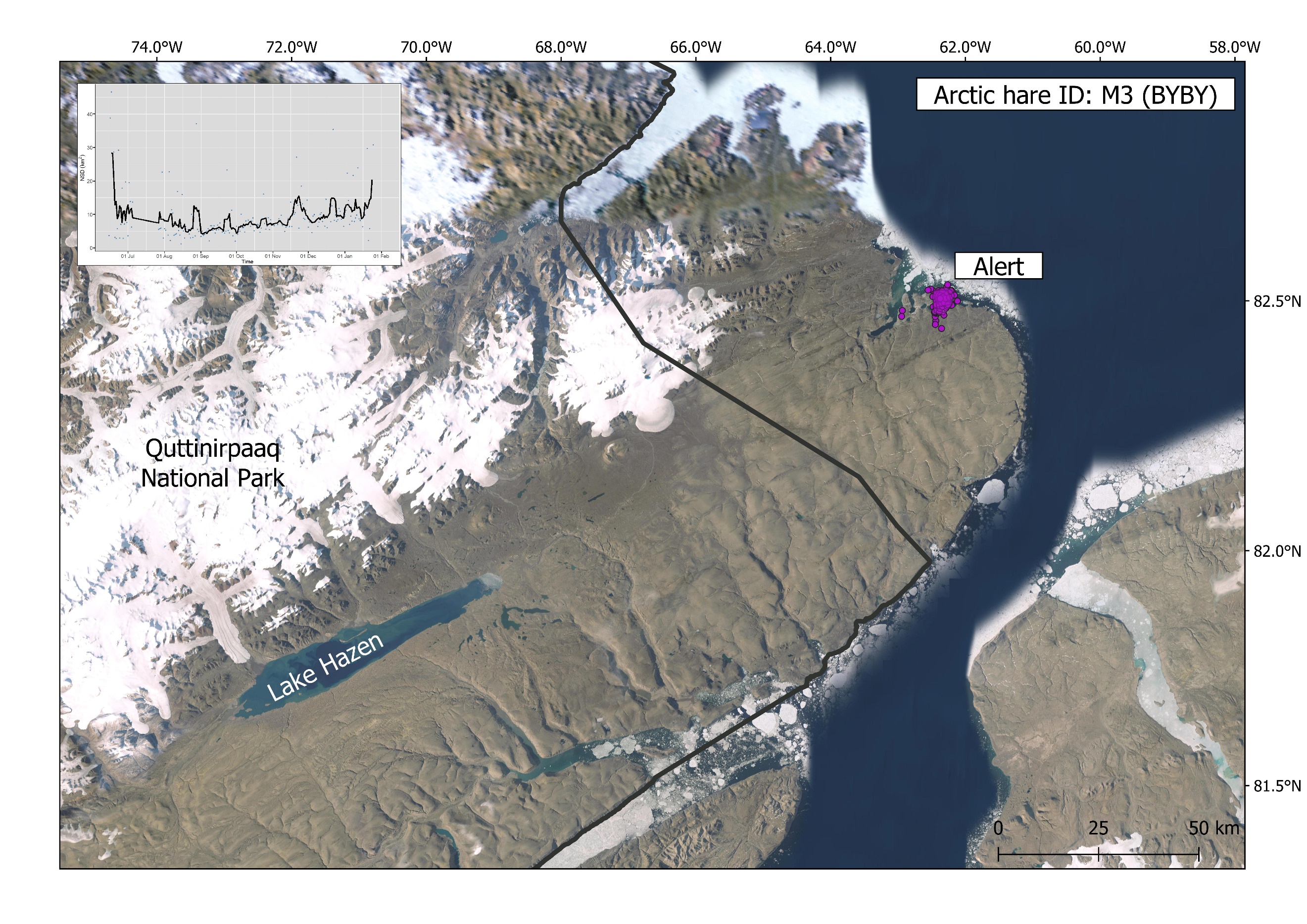


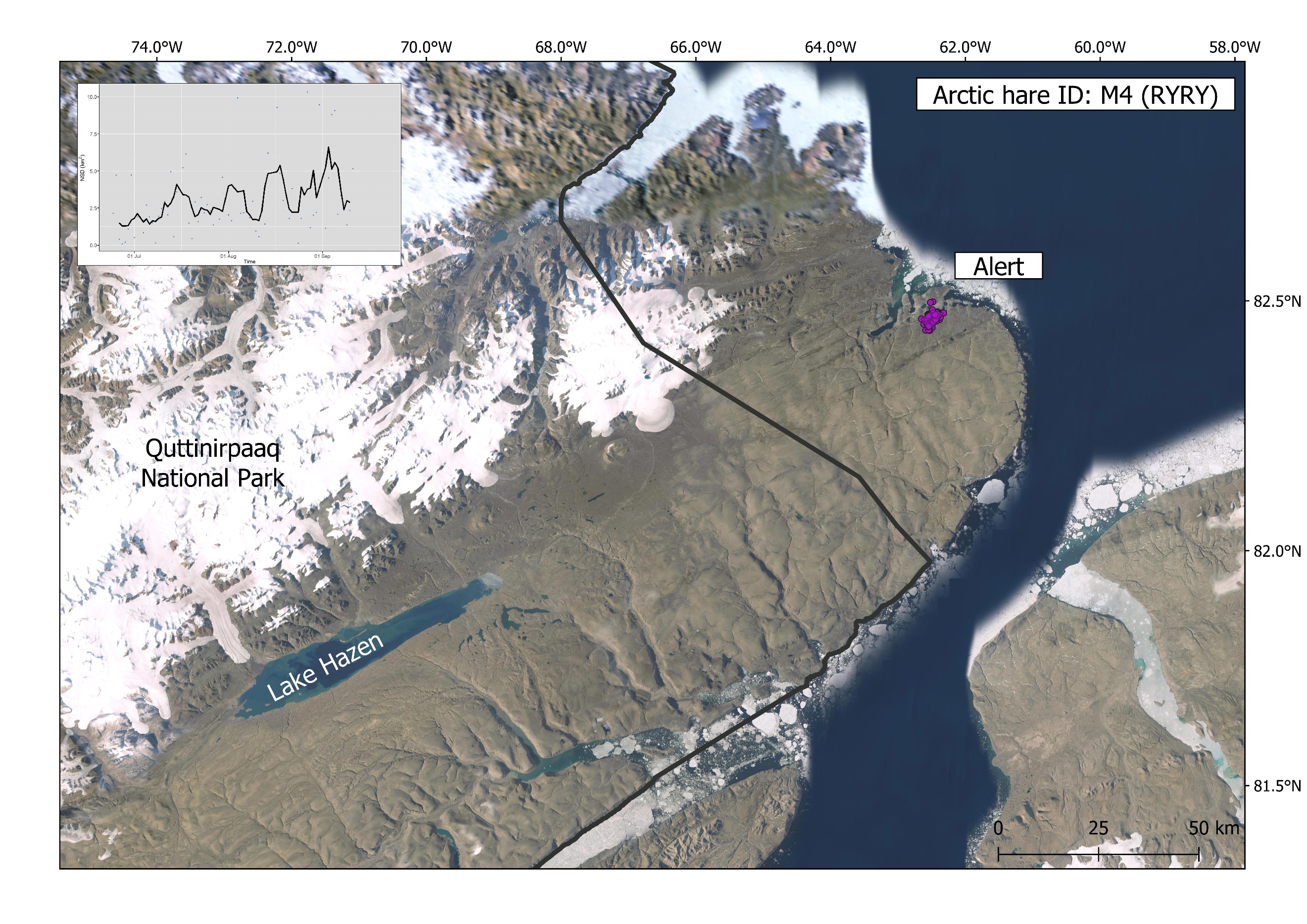

Supplement: Supplementary file 1 — Supplementary Figure S1. [file 41598_2022_8347_MOESM1_ESM.docx]
